# Buffer and Polymer Molecular Weight Affect Zinc Myoglobin-Mediated PET-RAFT Polymerizations

**DOI:** 10.1021/acsmacrolett.6c00164

**Published:** 2026-05-09

**Authors:** Ian C. Anderson, Mikayla R. Smith, Stephen J. Koehler, Baofu Qiao, C. Adrian Figg

**Affiliations:** † Department of Chemistry and Macromolecules Innovation Institute, 1757 Virginia Tech, Blacksburg, Virginia 24061, United States of America; ‡ Department of Natural Sciences, 5920 Baruch College, City University of New York, New York, New York 10010, United States of America

## Abstract

Protein photocatalysts provide a powerful and highly adaptable method for synthesizing and transforming small organic molecules; however, they remain underexplored for photocatalytic polymer synthesis. In particular, the effects of polymer molecular weight and protein structure are unknown in the context of photoinduced electron/energy transfer reversible-activation fragmentation chain transfer (PET-RAFT) polymerizations. Herein, we investigate the effects of buffer conditions and the molecular weights of macro chain-transfer agents (macro-CTA) on zinc myoglobin-mediated PET-RAFT chain-extension polymerizations. Chain extensions performed in tris buffer showed a 17% higher apparent rate constant than those performed in PBS. Macro-CTA molar mass also affected the rate of chain extension polymerizations, where a 22 kg/mol macro-CTA showed a chain extension apparent rate constant 40% faster than a 75 kg/mol macro-CTA. Finally, we calculated the solvent-accessible surface area (SASA) of model polymers and found that the RAFT end group becomes less accessible to the protein photocatalyst as the macro-CTA molar mass increases, corroborating the observed decrease in the apparent rate constant. Overall, this work provides insight into the use of proteins as PET-RAFT catalysts by examining how polymerization conditions affect catalyst performance.

## Graphical abstract

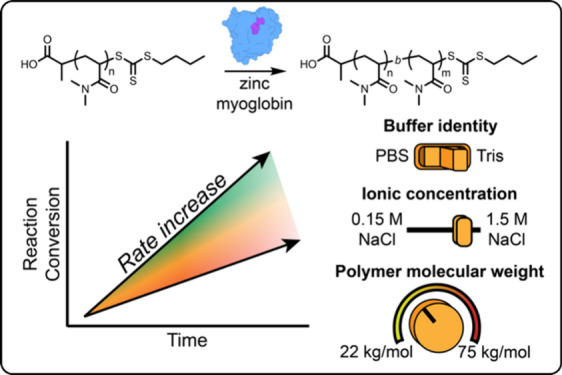

Reversible deactivation radical polymerization (RDRP) enables the synthesis of polymers with tunable molecular weights and narrow dispersities.^1,2^ RDRP methods can reliably synthesize polymers and block copolymers for applications such as polymerization-induced self-assembly,^3–6^ modulating protein function,^7^ surface patterning,^8–11^ and polymer blend compatibilizers.^12,13^ Photoinduced electron/energy transfer reversible addition–fragmentation chain transfer (PET-RAFT) polymerization, developed by Boyer and coworkers, is a type of RDRP that uses photocatalysts to cleave the C–S bond of a thiocarbonylthio compound and introduce radicals to a polymerization.^14–16^ Many different types of photocatalysts have been used for PET-RAFT polymerization, such as metal complexes,^17,18^ organic dyes,^19,20^ polymer nanoparticles,^10,21,22^ perovskites,^23–27^ metal organic frameworks (MOFS),^28–31^ naturally derived porphyrins,^32,33^ and biomolecules.^34^

Biomacromolecules have emerged as useful tools for a number of challenging organic transformations^35–39^ and for atom-transfer radical polymerizations (ATRP).^40^ For example, Bruns and coworkers have shown that horseradish peroxidase, hemoglobin, and red blood cells are effective ATRP catalysts and can be used for artificial cell synthesis.^41–43^ Photoactive proteins are less commonly used for polymer synthesis. However, An and coworkers used glucose oxidase to transfer electrons to monomers, generating a propagating radical for RAFT polymerization.^44^ Artificial metalloproteins, in which the native cofactor has been synthetically replaced with a different one, endow protein scaffolds with new reactivity, such as photocatalyzed electron transfer, enabled by the new non-native cofactor group.^35,45–47^

Recently, we reported the first example of using an inherently photoactive protein, zinc myoglobin (ZnMb), to conduct a PET-RAFT polymerization of acrylamides.^34^ In these experiments, we observed that the apparent rate constants of polymerization decreased 2× during the course of the polymerization. Additionally, the UV–vis absorbance of ZnMb decreased over time, suggesting degradation. In addition to protein degradation during polymerization, we expected that the apparent decrease in rate was due to steric hindrance between the growing polymer and the protein catalyst. While there is some understanding that protein catalysts can slow down reaction rates due to steric hindrance,^48^ this effect is not observed in RAFT polymerization because proteins are typically used as conventional initiation sources. In contrast, during PET-RAFT polymerization, the protein must directly interact with the polymer chain end to initiate propagating radical formation. Therefore, we hypothesized that the molecular weight of the growing polymer chain affects the apparent rate constants of ZnMb polymerizations due to the increased steric hindrance between the polymer and the protein.

To test the effect of molecular weight on chain-extension kinetics, we prepared two different poly­(*N*,*N*-dimethylacrylamide) (PDMA) macro chain-transfer agents (macro-CTAs) with molecular weights of 22 and 75 kg/mol. We then performed an extensive kinetic analysis of PET-RAFT chain extensions using each macro-CTA (Figure 1). Additionally, we expected that buffer conditions could be altered to improve protein stability. To probe the effect of buffer choice, we compared phosphate-buffered saline (PBS) to tris-buffered saline (tris) with increasing NaCl concentrations. Overall, these experiments show that protein catalysts can be stabilized using suitable buffer conditions and that steric effects are a critical consideration in protein–polymer interactions, particularly when the protein is being used as a catalyst for polymerization.

**Figure 1:**
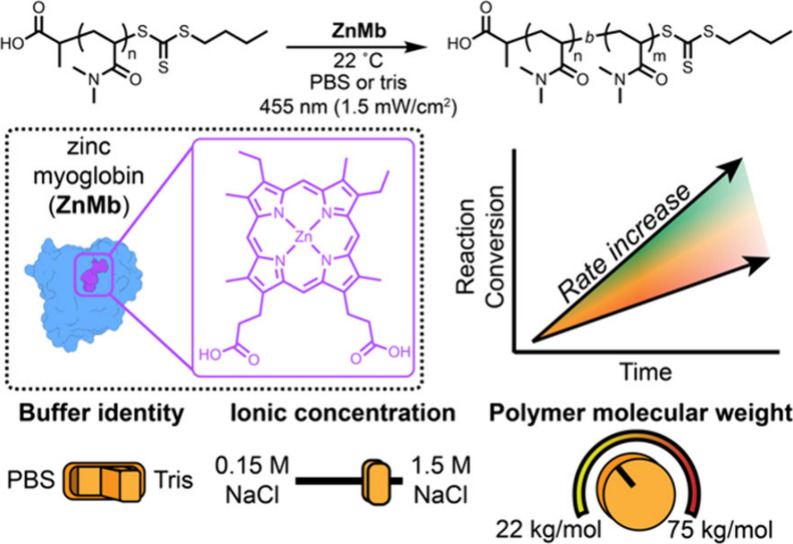
Analyzing the chain-extension polymerizations of poly­(*N*,*N*-dimethylacrylamide) of different molecular weights in different buffer conditions.

The two PDMA macro-CTAs were synthesized via thermal RAFT polymerization initiated by 4,4′-azobis­(4-cyanovaleric acid) (ACVA) at 75 °C using 2-(butylthiocarbonothioylthio) propanoic acid (BTPA) as the chain transfer agent. The polymerizations were run for 25 min, during which each reached approximately 60% conversion, as determined by ^1^H NMR spectroscopy (Figures S1 and S2). This conversion ensured the retention of the trithiocarbonate for subsequent chain-extension polymerization analysis. Following polymerization, the PDMA macro-CTAs were precipitated into cold ether, dried under vacuum overnight, and analyzed by gel permeation chromatography (GPC) to confirm the molar masses of the macro-CTAs (Figure S3).

To measure how buffer conditions affect the ZnMb-mediated PET-RAFT polymerizations under blue-light irradiation, the 22 kg/mol PDMA (*Đ* = 1.03) was used for chain-extension polymerizations. We selected this macro-CTA for these experiments to ensure that any steric effects from the larger macro-CTA would not confound the buffer measurements. All polymerizations used a macro-CTA:ZnMb loading of 1:0.01 (i.e., 6.25 × 10^–5^ mM ZnMb). Kinetic measurements were periodically taken on the ZnMb-catalyzed polymerization samples, which were analyzed by ^1^H NMR spectroscopy and GPC.

First, comparing PBS and tris with NaCl concentrations (0.14 and 0.15 M, respectively) that reflect standard buffer conditions showed distinct differences in the pseudo-first-order kinetics plot later in the polymerization (Figures 2a and S4–S7). The polymerization performed in tris showed linear kinetic behavior, as expected for a controlled polymerization. In contrast, polymerizations in PBS showed a similar decrease in the apparent rate constant during polymerization as we observed previously.^34^ Specifically, tris showed an apparent rate constant of 0.23 ± 0.02 h^–1^ for the polymerization compared to 0.19 ± 0.02 h^–1^ in PBS (Figures S18 and S19). However, number-average molecular weight (*M*_n_) versus conversion and dispersity plots showed little difference in the polymerization outcomes in PBS and tris (Figures 2b, S8, and S9; Tables S1 and S2). These results suggest that there are inherent differences in the capacity of the buffer to stabilize ZnMb, but these differences do not affect the polymer product.

**Figure 2:**
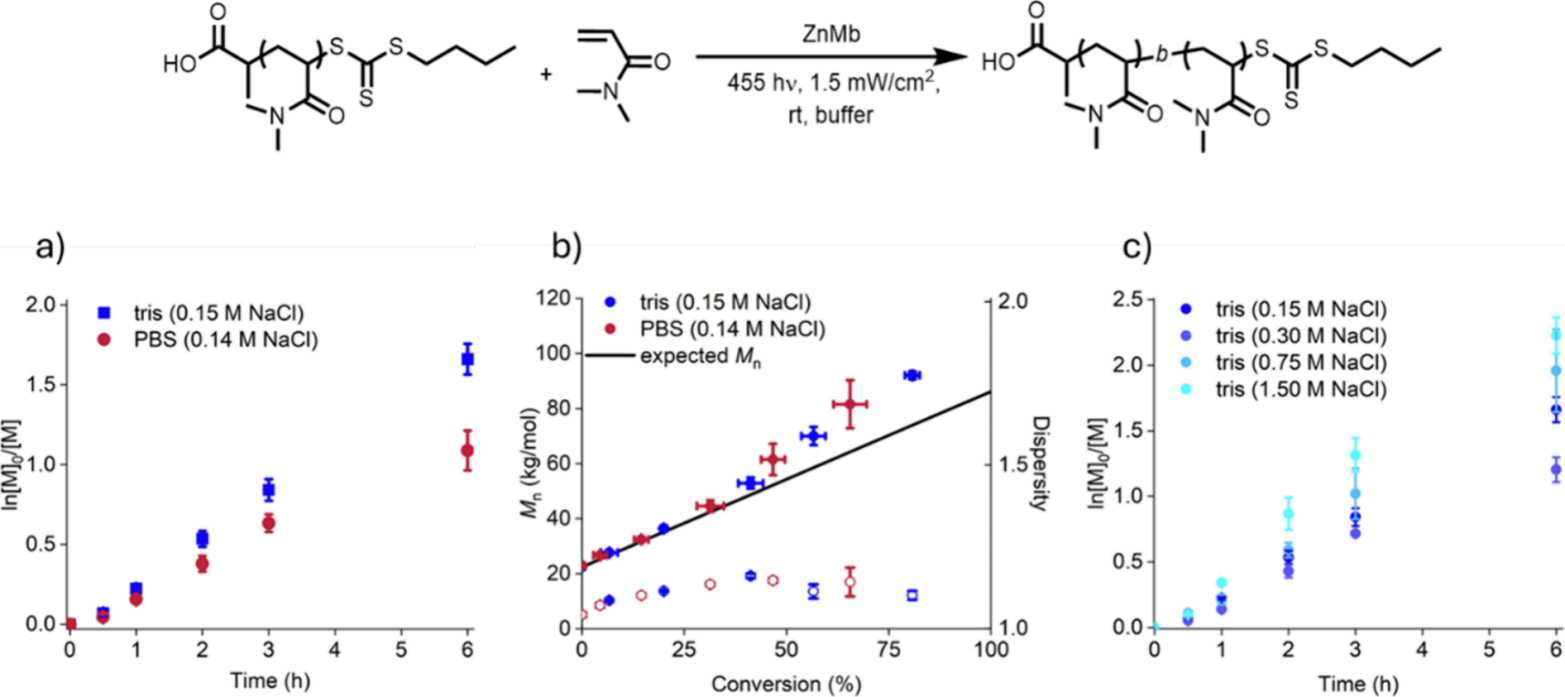
(a) Comparison of PET-RAFT polymerization kinetics using ZnMb between tris (0.15 M NaCl) and PBS (0.14 M NaCl) buffers with a 22 kg/mol macro-CTA. (b) Plot comparing the *M*_n_ of chain-extension polymerizations to the expected value and the dispersity values. (c) Polymerization kinetics in tris with different NaCl concentrations.

Tris has three hydroxyls, three methylenes, and one free amine. These structural features are known to help stabilize proteins, such as bovine serum albumin.^49^ However, the amine can react with the protein over time, so non-nucleophilic buffers such as PBS are better for long-term storage.^36^

Since ZnMb is used within days of synthesis, we expect that the denaturation results from protein–polymer interactions instead of protein-buffer interactions. Therefore, the greater amphiphilicity of tris likely helps shield ZnMb from nonspecific aliphatic interactions with PDMA, which could otherwise denature the protein. Tris could also act as a sacrificial reducing agent, slightly increasing the polymerization kinetics. However, tris is typically a very poor reducing agent because of its high oxidation potential.^50^

Next, the NaCl concentration in the tris was adjusted to examine how ionic concentration affects polymerization kinetics. Chain extension polymerization kinetics using the 22 kg/mol macro-CTA were performed in tris with 0.15 (standard tris), 0.30, 0.75, or 1.5 M NaCl. The kinetics showed that changing buffer conditions affected the polymerization rate (Figures 2c and S10–S13). Specifically, increasing the NaCl concentration in the polymerization mixture slightly increased the polymerization apparent rate constant from 0.28 ± 0.02 h^–1^ to 0.39 ± 0.03 h^–1^. Regardless of the buffer choice, the polymerization initially exhibited linear pseudo-first-order kinetics. A slight downward curvature in the kinetics plots was observed at higher conversions, indicating that termination events were not entirely prevented, but this effect was greatly reduced at higher salt concentrations than at standard buffer NaCl concentrations. All conditions also produced a linear increase in molecular weight with conversion, although later time points showed higher-than-expected molecular weights. These results confirmed that tris with higher ionic concentrations led to faster ZnMb-mediated PET-RAFT polymerizations. We initially expected that higher ionic content would stabilize ZnMb, but minimal changes to protein structure were detected by circular dichroism (Figure S14). The higher salt concentration may be affecting polymer–protein interactions or slightly stabilizing the porphyrin within the protein, but further experimental and computational experiments are warranted.

To test how the macro-CTA molecular weight affected ZnMb-mediated PET-RAFT polymerizations, kinetic measurements were performed comparing the 22 kg/mol PDMA to the 75 kg/mol PDMA (*Đ* = 1.09) in tris with either 0.15 or 1.5 M NaCl. In both buffer conditions, the 75 kg/mol PDMA macro-CTA showed a slower apparent rate of polymerization compared to the 22 kg/mol macro-CTA (Figures 3a, S14, and S15). The apparent rate constant decreased from 0.28 ± 0.02 h^–1^ to 0.21 ± 0.02 h^–1^ going from a 22 kg/mol to 75 kg/mol macro-CTA in tris (0.15 M NaCl) and from 0.39 ± 0.03 h^–1^ to 0.23 ± 0.05 h^–1^ going from a 22 kg/mol to 75 kg/mol macro-CTA in tris (1.5 M NaCl) (Figures 3b, S16, and S17). The 75 kg/mol macro-CTA with 0.15 M NaCl showed evidence of slower initiation than with 1.5 M NaCl, further suggesting that a combination of the buffer composition and polymer molecular weight affects polymerization kinetics.

**Figure 3:**
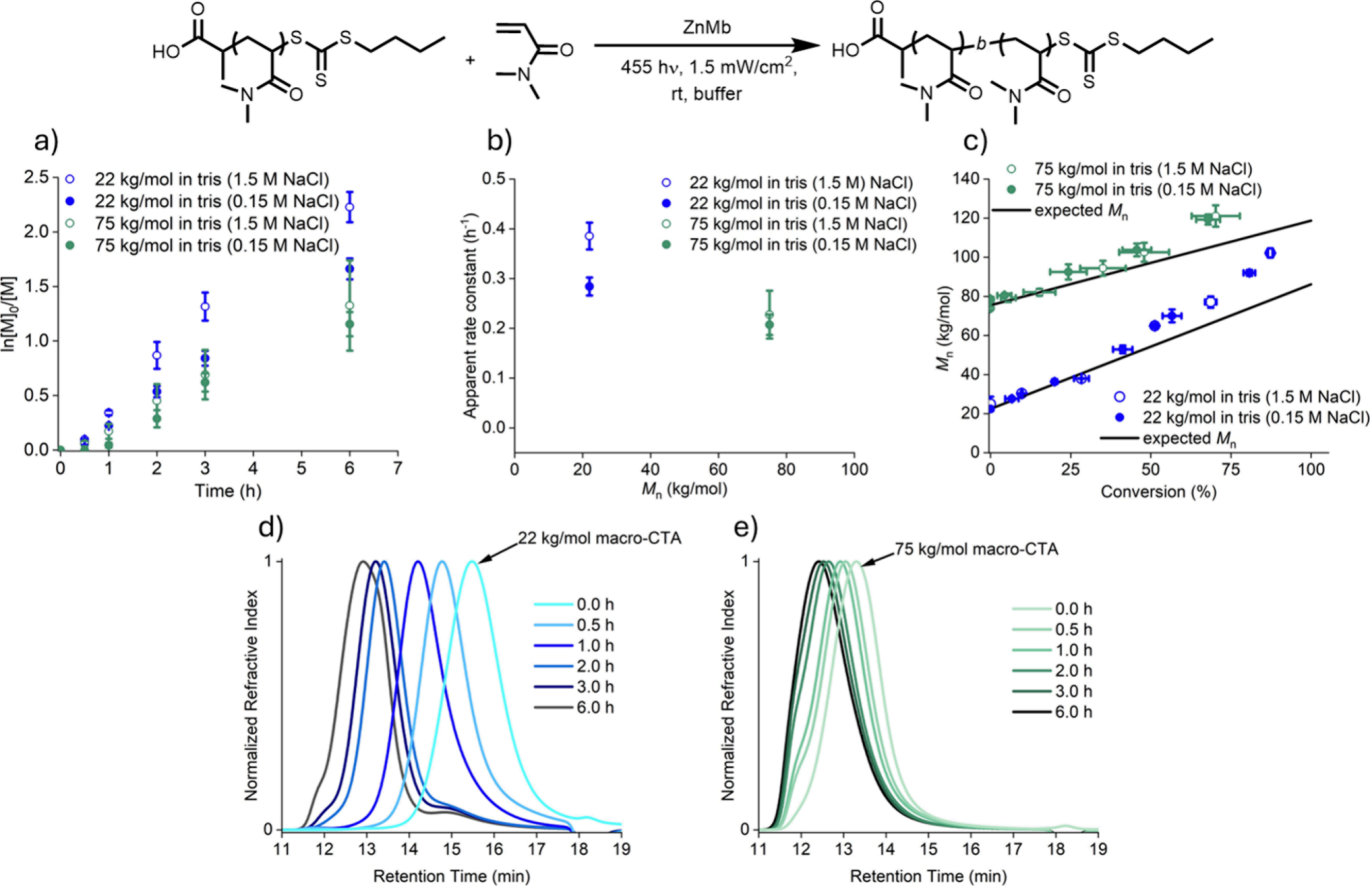
(a) Pseudo-first-order kinetics covering both sets of macro-CTAs in tris 0.15 and 1.5 M NaCl buffer conditions. (b) Apparent rate constants for different PDMA macro-CTA molecular weights and salt concentrations. (c) *M*_n_ vs conversion for different macro-CTA and salt concentrations. (d) GPC traces of polymerization aliquots for the 22 kg/mol macro-CTA in tris (1.5 M NaCl). (e) GPC traces when the 75 kg/mol macro-CTA in tris (1.5 M NaCl).

GPC analysis was performed to determine how the molecular weight of the macro-CTA affected the products of the chain-extension reactions. When analyzing the conversion versus *M*_n_ data (Figure 3c; Tables S3–S5), polymerizations with either macro-CTA showed linear increases early in the polymerization. However, data showed that using a lower-molecular-weight macro-CTA led to greater deviation from the expected block copolymer molecular weight later in the polymerization, regardless of the NaCl concentration. The deviation may result from termination events that reduce the number of growing polymer chains relative to the monomer concentration, yielding higher-than-expected molecular weights.^51^

The chromatograms resulting from the GPC analysis of the 22 kg/mol macro-CTA uniformly shifted to lower retention times early in the polymerization (Figures 3d and S18). However, the data also suggested that termination events were occurring later in the chain extension, as evidenced by multimodal traces at later time points (e.g., time point 2 h), but these accounted for less than 5% of total chains. The chromatograms from the 75 kg/mol macro-CTA chain extension polymerization suggested fewer termination events at low molecular weights (Figures 3e, S19, and S20), but the exclusion limit of the column precluded analysis of any chain–chain coupling termination events. Overall, these data indicated that while ionic concentration may help stabilize ZnMb during PET-RAFT polymerizations, the apparent rate constants decreased with increasing polymer molecular weight.

To better understand the relationship between ZnMb and the sizes of different macro-CTAs, we calculated the exposure of the macro-CTAs with varying molecular weights by means of all-atom explicit solvent molecular dynamics simulations. Specifically, the Solvent Accessible Surface Areas (SASAs) of the polymer chains and their end groups were calculated. SASA estimates the surface of a molecule that is accessible to a probe of a defined size. We compared a water-sized probe (radius = 0.14 nm) to a probe of the size of ZnMb (radius = 1.75 nm). PDMA with degrees of polymerization (DPs) of 50, 100, 200, 400, and 600 was simulated using all-atom explicit-solvent molecular dynamics simulations (Figure S21). Following PDMA equilibration, the water or ZnMb-mimic probes were used to determine both the overall polymer SASA and the chain-end SASA of the trithiocarbonate. The polymer SASA decreased linearly with increasing DP (Table S6) with either probe, showing that the polymer chain became more compact. In contrast, when analyzing the SASA for the chain end with the RAFT agent (Figure 4, Table S6), there was a noticeable drop going from DP 100 (SASA = 10.4 ± 2.1 nm^2^) to 200 (SASA = 3.8 ± 2.4 nm^2^). These results suggested that, as the *M*_n_ of PDMA exceeds 10 kg/mol, the trithiocarbonate became less accessible to ZnMb catalysts. The less-accessible chain end would lead to slower polymerization kinetics, which is consistent with the change in apparent rate constants we observed as we increased the macro-CTA *M*_n_.

**Figure 4:**
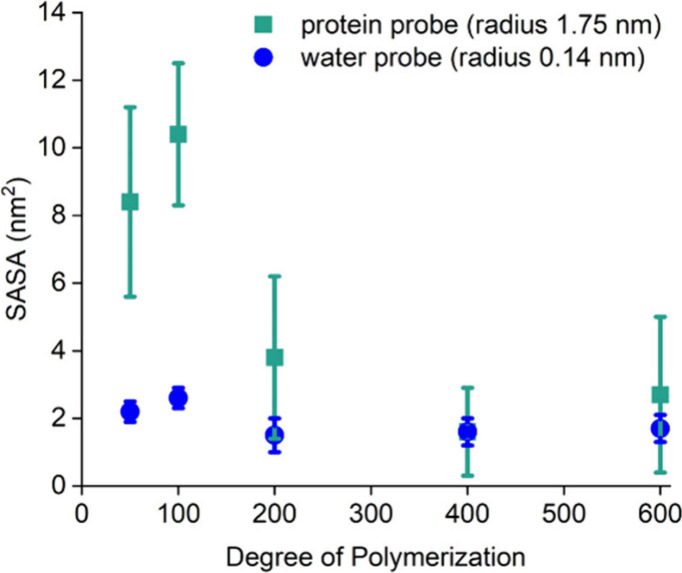
SASA of the RAFT chain ends when a protein-sized probe or a smaller, water-sized probe is used. The errors stand for the standard deviations from three parallel runs.

The effects of different chain extension conditions were studied using ZnMb as the catalyst for PET-RAFT polymerizations. First, buffer conditions affect protein stability and the resulting polymerization kinetic behavior. Second, experimental and computational data support the hypothesis that the molecular weight of the polymer affects the apparent rate constants of chain extension from macro-CTAs when proteins are used as photocatalysts in PET-RAFT polymerizations. Overall, we identified two key parameters that can be tuned to access more defined polymer structures when using proteins as catalysts for polymer synthesis. These results provide critical insights into guiding directed evolution approaches toward achieving selective, highly active protein photocatalysts for PET-RAFT polymerizations.

## Supplementary Material



## Notes

Experimental and simulation methods. Supporting figures and supporting tables (PDF)
