# Supplementary material for: Buffer and Polymer Molecular Weight Affect Zinc Myoglobin-Mediated PET-RAFT Polymerizations
Source: ACS Macro Lett. 2026 May 9;15(5):732–7. doi: 10.1021/acsmacrolett.6c00164 (PMC13192322; doi:10.1021/acsmacrolett.6c00164)
Supplement: Supplementary file 1 [file mz6c00164_si_001.pdf]

Supporting Information for

Buffer and Polymer Molecular Weight Affect Zinc Myoglobin-Mediated PET-RAFT Polymerizations

Ian C. Anderson,[a] Mikayla R. Smith,[a] Stephen J. Koehler,[a] Baofu Qiao,*[b] and C. Adrian Figg*[a]

[a] Department of Chemistry and Macromolecules Innovation Institute, Virginia Tech, Blacksburg, Virginia, 24061, United States of America

[b] Department of Natural Sciences, Baruch College, City University of New York, New York, New York, 10010, United States of America

Table of Contents

Experimental:......1

Materials:......2

Analysis:......2

Procedures:......3

All-atom explicit solvent molecular dynamics (MD) simulations:......7

Supporting Figures:......10

Supporting Tables:......41

References:......44

Experimental:

Materials:

Myoglobin (from equine skeletal muscle 95-100%, lyophilized powder), mesoporphyrin IX (95%), and were purchased from Sigma Aldrich and used as received. *N*,*N*-Dimethylacrylamide (DMA, >99%) was filtered through basic alumina prior to use. Solvents were purchased from Thermo Fisher; buffer pH was monitored using a Thermo Fisher Orion Star A111 pH meter. ZnMIX was synthesized using the reported methods[1, 2] and the purity was assessed by thin-layer chromatography and UV-Vis spectroscopy. 4,4`-azobis(4-cyanovaleric acid) (ACVA, Sigma Aldrich, >98%) was prepared as a 2 mg/mL solution in reaction solvent prior to use. N,N-dimethylacetamide (Thermo Scientific, 99%), and N,N-dimethylformamide (Acros Organics, 99.8%) were used as received. 2- (Butylthiocarbonothioylthio)propionic acid (BTPA) was synthesized according to a previous report. [3]

For a visible light source, 76.2 cm Supernight Blue Light Strips were purchased from Amazon and placed on a Xnrtop Silver Tone Aluminum Radiator Heat Sink 150 × 80 × 27 mm from Amazon.

Analysis:

^{1}H NMR spectroscopy was conducted on either an Agilent U4-DD2 400 MHz or a Jeol VH 400 MHz. Samples were prepared with D_2O (Cambridge Isotopes Laboratories, Inc., 99.9%) or DMSO-d_6 (Cambridge Isotopes Laboratories, Inc., 99.8%), which were used as received. Conversion was calculated by the equation $Conv\ \% = \frac{[DMA_o]-[DMA_t]}{[DMA_o]} \times 100$

Size Exclusion Chromatography (SEC) was performed in 1x PBS with 200 mg/L NaN_3 at a flow rate of 0.5 mL min^{-1} (Agilent isocratic pump, degasser, and autosampler, columns: TOSOH TSKgel Guard PW_{XL} and TOSOH TSKgel G3000PW_{XL} molecular weight range 0-1 ×

10^5 g mol^{-1}. Detection consisted of a Wyatt Optilab refractive index detector operating at 785 nm, a Wyatt DAWN multi-angle light scattering detector operating at 783 nm, and an Agilent MWD operating at 365 nm. Absolute molecular weights and dispersities were calculated with the Wyatt ASTRA software. d*n*/d*c* values were calculated using the d*n*/d*c* from the mass recovery method built into the Wyatt ASTRA software. A d*n*/d*c* of 0.1409 was calculated for the 22 kg/mol macro-CTA, and a d*n*/d*c* value of 0.1432 was calculated for the 75 kg/mol macro-CTA. Those values were used to calculate the masses for the respective polymerization aliquots.

Visible light intensity was measured with an International Light Technologies ILT-350 illuminance spectrophotometer with a NIST traceable ISO17025 accredited calibration.

Circular Dichroism (CD) spectroscopy was performed on a Jasco J-815 CD spectrometer using a 1 mm pathlength Jasco quartz cuvette at room temperature. Samples were prepared at a protein concentration of 0.25 mg/mL in with NaCl concentrations of PBS (0.14, 0.28 0.70, or 1.4 M) or tris (0.15, 0.30, 0.75, or 1.5M) buffer as described.

UV-Vis spectroscopy was performed on a Cary 60 UV-Vis using 1 cm quartz cuvettes

Procedures:

Zinc myoglobin synthesis

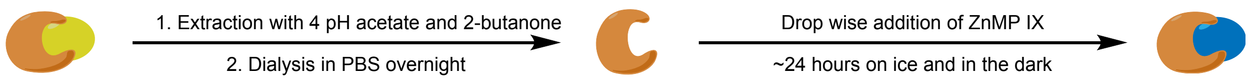

Apo myoglobin was prepared using a modified reported method.[61,105] Myoglobin (25 mg) was added to a 50 mL centrifuge tube and dissolved into 25 mL of acetate buffer chilled at 4 °C (pH 4) to yield a 1.0 mg/mL solution. An equal volume of butanone chilled at 4 °C (25 mL) was added to the tube and agitated to denature myoglobin and extract the heme group the top organic layer was removed. This extraction process was repeated 3× until the organic layer was colorless.

The solution was diluted 4× with PBS chilled at 4 °C, put into a 6-8 kDa dialysis bag (Spectra/Por 1 Dialysis Membrane Standard RC Tubing, SpectrumLabs Inc), and dialyzed against 5.0 L of PBS changing the dialysis buffer 3×. The solution was collected and centrifuged to remove any insoluble precipitates. The solution containing apo-myoglobin was concentrated using spin filtration with a 10 kDa MWCO spin filter (Pierce Protein Concentrator PES), until the volume was 100 mL. Separately, a solution of ZnMIX was prepared in PBS with 15 v/v% DMF using 6:1 molar equivalents of ZnMIX:apo myoglobin (determined using the 280 nm absorption peak, **ε**=13,980 L mol^{-1} cm^{-1}).[111] The ZnMIX solution was added dropwise to the apo myoglobin solution overnight in an ice bath. The ZnMb solution was purified using both CM 52 and G 25 size exclusion columns, concentrated using a 10 kDa MWCO spin filter (Pierce, Thermo Fisher) until the ZnMb concentration reached >3.0 mg/mL by UV-Vis spectroscopy (280 nm, **ε**= 13,980 ZnMb L mol^{-1} cm^{-1}). Solutions were used within 7 days of synthesis.

22 kg/mol Macro CTA Synthesis

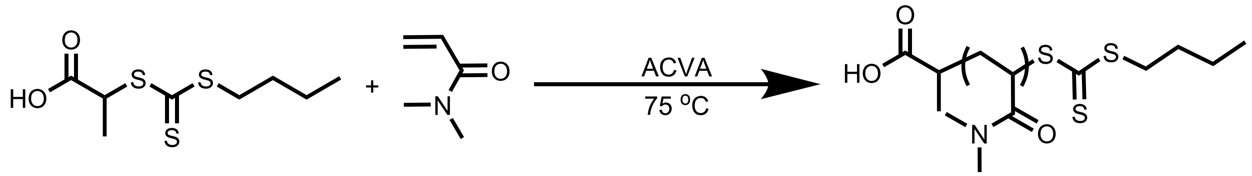

BTPA (173 mg, 0.73 mmol) was dissolved in a 2 dram vial with 7 mL of DMAc. This solution was added to a 50 mL Schlenk flask with *N*,*N*-dimethylacrylamide (19.2 g, 200 mmol) and 1.32 mL of an ACVA solution (3.1 mg/mL) in DMAc (4.1 mg, 0.015 mmol) with 1-3 drops of DMF as an internal standard. The volume was adjusted up 40 mL total with DMAc. The reaction mixture was bubbled with argon for 40 minutes and then heated at 75 °C for 25 minutes. The reaction was then stopped by opening the flask to air and removing it from the oil bath. Conversion

was determined by ^{1}H NMR spectroscopy using $Conv\,\% = \frac{[DMA_o]-[DMA_t]}{[DMA_o]} \times 100$. The polymer was then precipitated into cold ether 3× and then dried under vacuum overnight. The dried polymer was used for SEC-MALLS analysis

75 kg/mol Macro CTA Synthesis

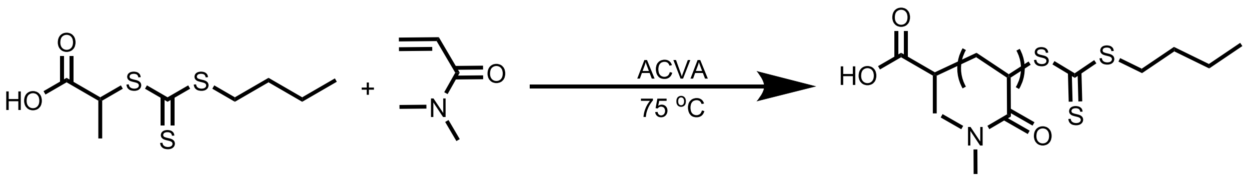

BTPA (38 mg, 0.16 mmol) was dissolved in a 2 dram vial with 7 mL of DMAc. This solution was added to a 50 mL Schlenk flask with *N*,*N*-dimethylacrylamide (19.2 g, 200 mmol) and 0.455 mL of an ACVA solution (1.97 mg/mL) in DMAc (0.896 mg, 0.003 mmol) with 1-3 drops of DMF as an internal standard. The volume was adjusted up 40 mL total with DMAc. The reaction mixture was bubbled with argon for 40 minutes and then heated at 75 °C for 25 minutes. The reaction was then stopped by opening the flask to air and removing it from the oil bath. Conversion was determined by ^{1}H NMR spectroscopy using $Conv\,\% = \frac{[DMA_o]-[DMA_t]}{[DMA_o]} \times 100$. The polymer was then precipitated into cold ether 3× and then dried under vacuum overnight. The dried polymer was used for SEC-MALLS analysis

Procedure for PDMA macro-CTA kinetics polymerizations with a 22 kg/mol polymer

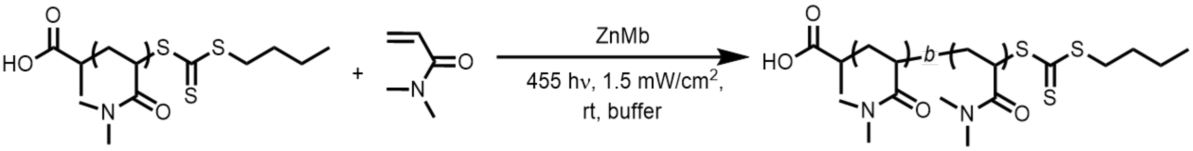

PDMA macro-CTA (87 mg, 0.00625 mmol) was dissolved in a 2 dram vial in 2.0 mL of buffer using a heated sonicator bath. This solution was added to a 10 mL Schlenk flask containing

DMA (257 μL, 2.5 mmol), ZnMb (6.25×10^{-5} mmol, variable concentration) in PBS or tris, and 1-3 drops of DMF as an internal standard. The NaCl concentration was raised to the desired level by spiking the solution with an appropriate volume of 5 M NaCl stock solution. The reaction volume was then raised to 5.0 mL using buffer so that the [DMA] = 0.5 M. The solution was bubbled with argon for 6 min, then irradiated with blue LED lights. Aliquots were periodically removed for ^{1}H NMR spectroscopy and SEC analysis. Conversion was calculated by the equation $Conv\,\% = \frac{[DMA_o]-[DMA_t]}{[DMA_o]} \times 100$.

Procedure for PDMA macro-CTA kinetics polymerizations with a 75 kg/mol polymer

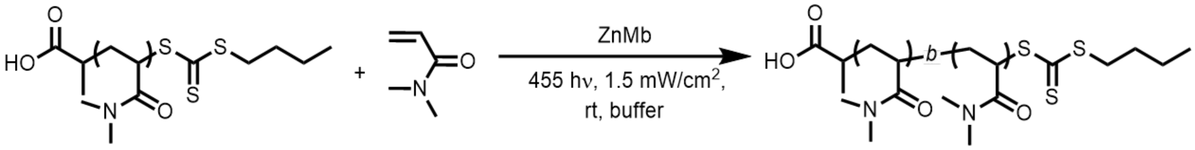

PDMA macro-CTA (432 mg, 0.00625 mmol) was dissolved in a 2 dram vial in 2.0 mL of buffer using a heated sonication bath. This solution was added to a 10 mL Schlenk flask containing DMA (257 μL, 2.5 mmol), ZnMb (6.25×10^{-5} mmol, variable concentration) in tris and 1-3 drops of DMF as an internal standard. The NaCl concentration was raised to the desired level by spiking the solution with an appropriate volume of 5 M NaCl stock solution. The reaction volume was then raised to 5.0 mL using buffer so that the [DMA] = 0.5 M. The solution was bubbled with argon for 6 min, then irradiated with blue LED lights. Aliquots were periodically removed for ^{1}H NMR spectroscopy and SEC analysis. Conversion was calculated by the equation $Conv\,\% = \frac{[DMA_o]-[DMA_t]}{[DMA_o]} \times 100$.

All-atom explicit solvent molecular dynamics (MD) simulations:

All-atom MD simulations were carried out to examine the structures of the polymers and the exposure of their tail groups. The open-source package GROMACS (version 2023) was employed.[4] The CGenFF (CHARMM general force field) potential[5, 6] was generated for the polymers using the CHARMM-GUI web server. [7] The recommended CHARMM TIP3P water model [8] was used with the structures constrained using the SETTLE algorithm. [9]

Guided by the experimental observation that these polymers are water-soluble, we simulated a single polymer chain dissolved in an aqueous solution. A fully extended polymer chain was created using an in-house script, which was equilibrated in a vacuum and collapsed within 100 ps. The polymer chain was then dissolved in a water box with an edge length of 8 nm for the polymer chain with the degree of polymerization DP = 50 and 100, and a larger length of 10 nm for longer polymer chains with DP = 200, 400, and 600. A salt concentration of 0.14 M NaCl was employed to mimic the PBS buffer in the experiments.[10] Given the fact that both the head and the tail groups of the polymers are negatively charged, two additional Na^+ ions were introduced to neutralize the whole system.

The energy minimization was first employed to equilibrate the system using the steepest descent algorithm, followed by further equilibrations using the canonical ensemble (constant number of particles, volume, and temperature, NVT) for a duration of 1 ps and the isothermal-isobaric ensemble (constant number of particles, pressure, and temperature, NPT) for a duration of 10 ps. Given the long chains of the polymers, their relaxation is known to be very slow, making it highly challenging for all-atom explicit solvent simulations.[10, 11] In this regard, the annealing simulation approach [10] (**Fig. S21**) was employed here. Four (or three for the system with the shortest polymer DP = 50) annealing cycles were conducted, with each cycle lasting 200 ns. For each annealing

cycle, the system temperature was increased from 298 K to 500 K within 10 ns, stayed at 500 K for 90 ns, decreased to 298 K within 10 ns, and stayed at 298 K for another 90 ns.

To further alleviate the impact of the strong correlation of the polymer chains owing to the slow relaxation, three parallel runs were carried out for each system based on the annealing simulation structures at 400 ns, 600 ns, and 800 ns (200 ns, 400 ns, and 600 ns for the system with DP = 50).[12] See **Fig. S21**. Each parallel run lasted for a duration of 200 ns for data collection and analysis. The neighbor searching was calculated up to a cutoff distance of 12 Å via the Verlet particle-based algorithm and was updated every 20th timestep. The short-range Coulomb interactions were truncated at the cutoff distance of 12 Å with the long-range interactions calculated using the Smooth Particle Mesh Ewald (PME) algorithm.[13, 14] The Lennard-Jones (LJ) 12-6 interactions were switched off from 10 Å to 12 Å via the potential-switch method. The temperature coupling was employed using the Nose-Hoover algorithm with the temperatures of water and non-water molecules separately coupled at 298 K with a characteristic time of 1 ps. The pressure was managed using the C-rescale algorithm at the reference pressure of 1 bar with a compressibility of 4.5×10^{-5} bar^{-1} and a constant of 5.0 ps. The integration time step of 2 fs was used with all the hydrogen-involved covalent bonds constrained using the LINCS algorithm.[15, 16] These parameters were recommended for the accurate reproduction of the original CHARMM simulation on lipid membranes[7] and have been verified in our simulations on polymers, [11, 17-22], proteins, [12, 23, 24] and more. The simulations were saved every 1 ns for data analysis. The initial and the final structures are presented in the inset of **Fig. S21** for the system with polymer DP = 100.

The solvent accessible surface area (SASA) was calculated using the following GROMACS program:

- *gmx sasa -f md.xtc -s md.tpr -n index -surface "polymer" -output "tail" -ndots 4800 -nopbc -probe 1.75 -o SASA_proteinProbe.xvg*
- *gmx sasa -f md.xtc -s md.tpr -n index -surface "polymer" -output "tail" -ndots 4800 -nopbc -probe 0.14 -o SASA_waterProbe.xvg*

Supporting Figures:

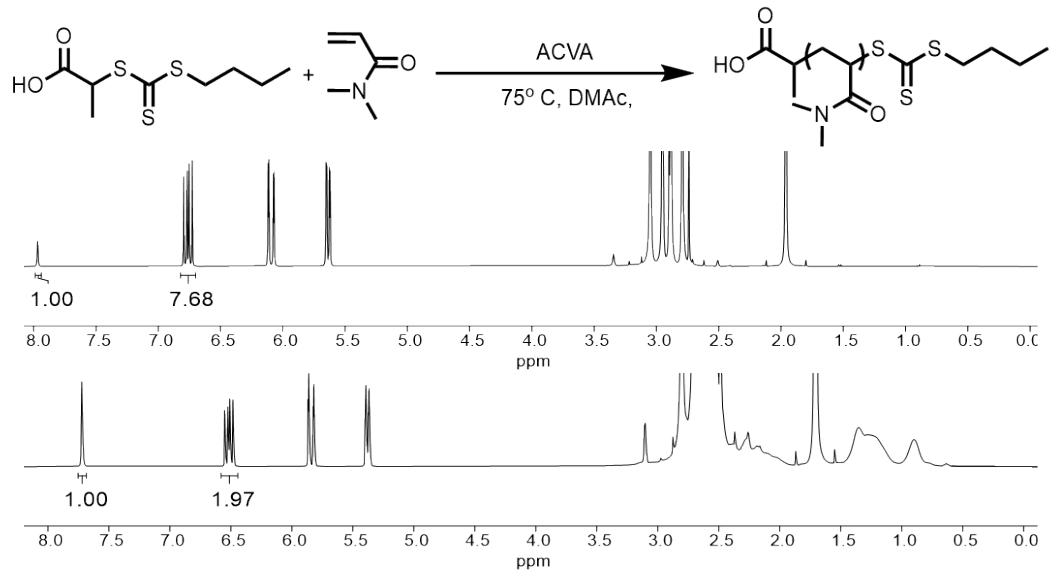

Figure S1. ^{1}H NMR spectra of 22 kg/mol macro-CTA synthesis used for kinetic experiments monitored by the disappearance of a DMA vinyl proton (δ=6.5 ppm) compared to a DMF internal standard aldehyde proton at (δ=7.8 ppm).

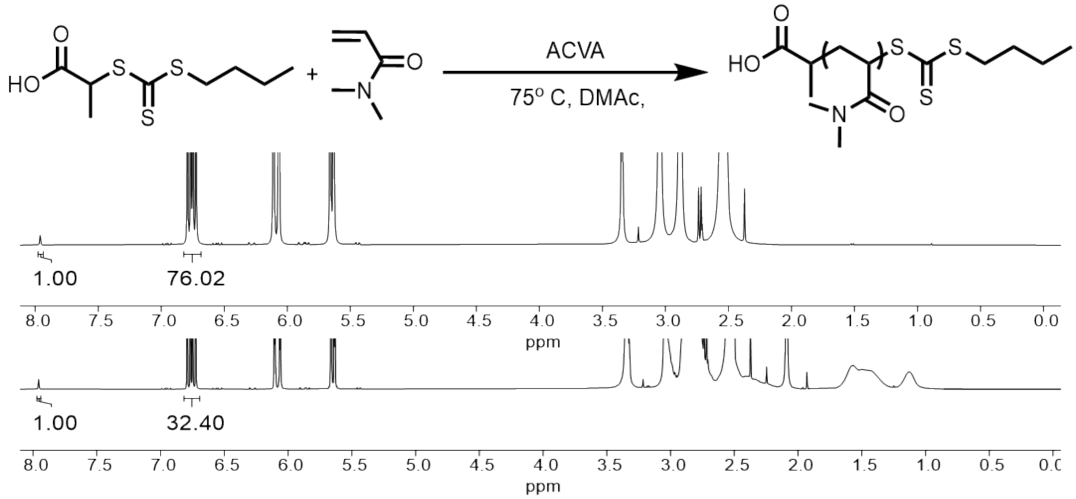

Figure S2. ^{1}H NMR spectra of 75 kg/mol macro-CTA synthesis used for kinetic experiments monitored by the disappearance of a DMA vinyl proton (δ=6.5 ppm) compared to a DMF internal standard aldehyde proton at (δ=7.8 ppm).

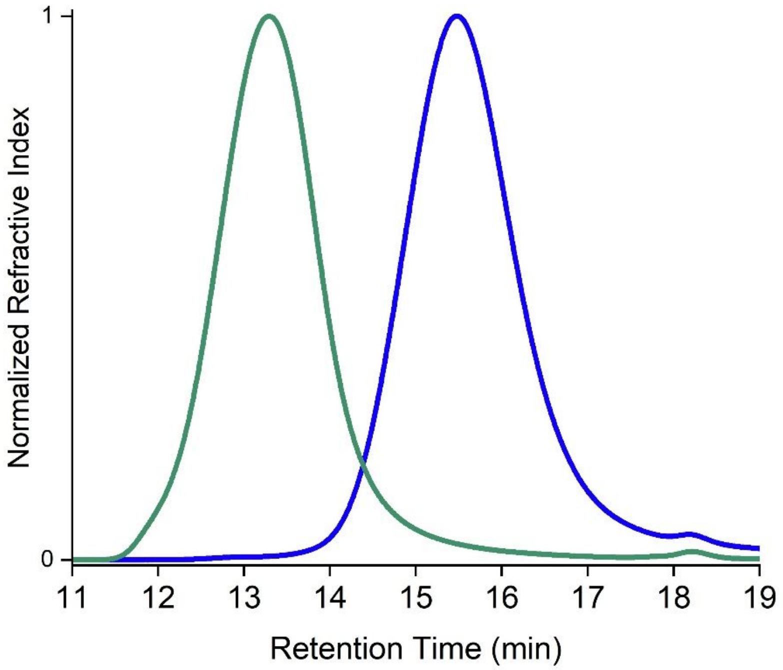

Figure S3. Normalized refractive index traces of 22 and 75 kg/mol macro-CTAs used for kinetic experiments. Dispersity values were determined to be 1.03 and 1.09, respectively. d*n*/d*c* values were calculated using the d*n*/d*c* from mass recovery method built into the Wyatt ASTRA software. A d*n*/d*c* of 0.1409 was calculated for the 22 kg/mol macro-CTA, and a d*n*/d*c* value of 0.1432 was calculated for the 75kg/mol macro-CTA. Those values were used to calculate the masses for the respective polymerization aliquots.

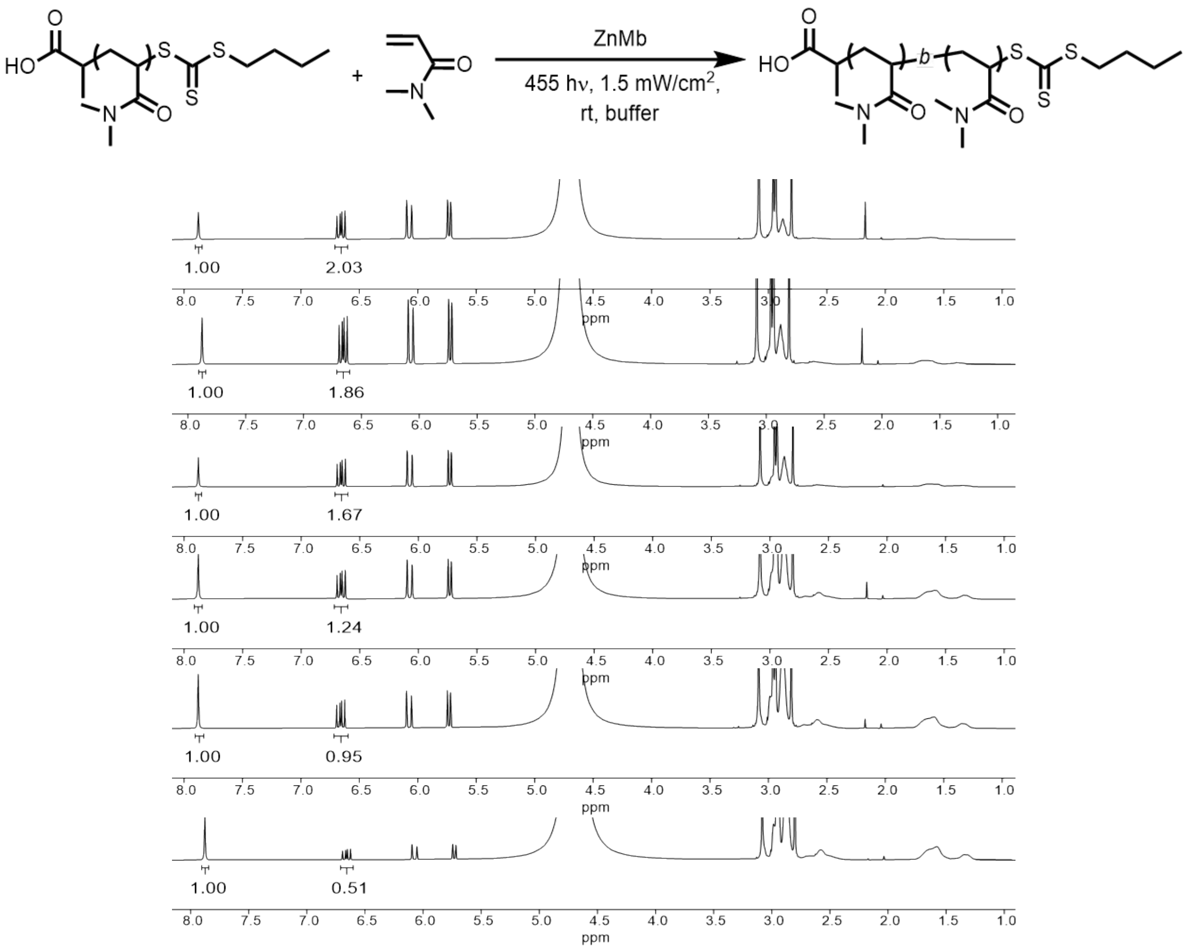

Trial 1

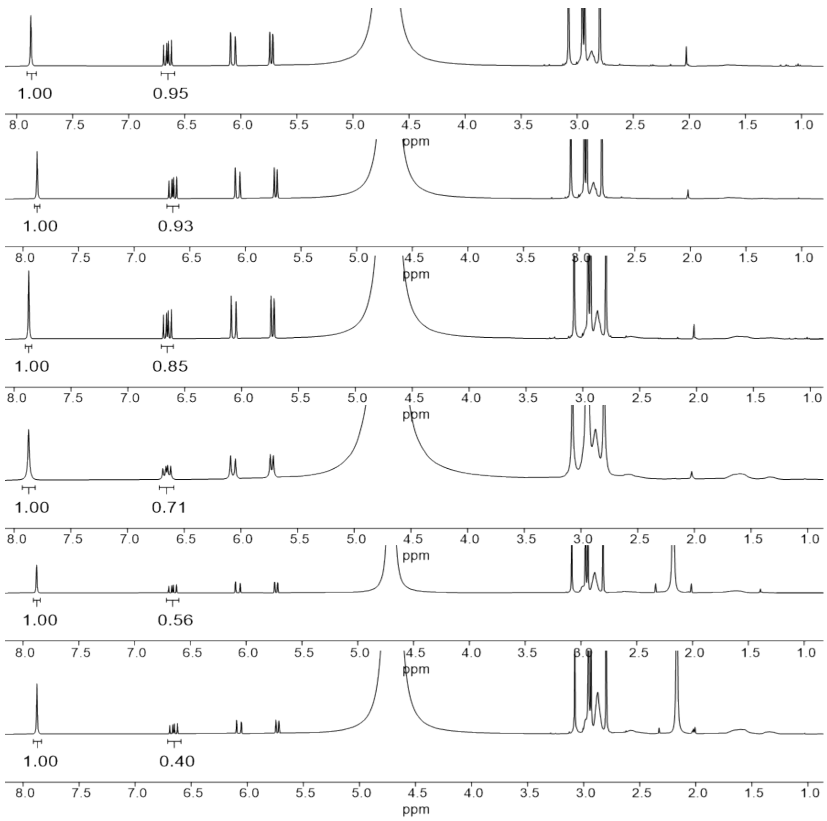

Trial 2

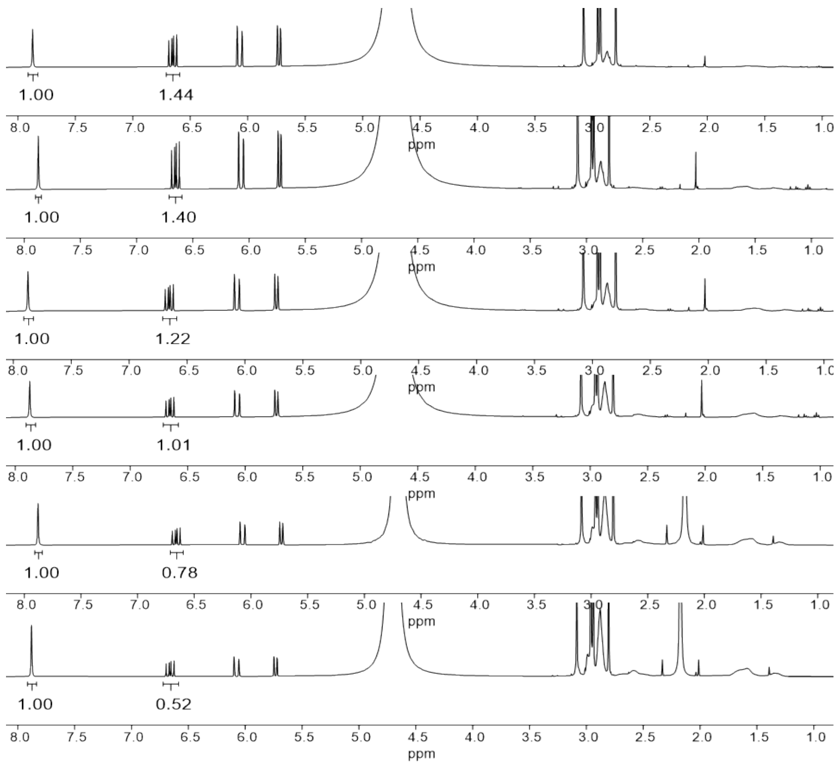

Trial 3

Figure S4. ^{1}H NMR spectra of kinetics for the replicates for PET-RAFT chain extension polymerization of a 22 kg/mol PDMA macro-CTA using zinc myoglobin in PBS (0.14 M NaCl) monitored by the disappearance of a DMA vinyl proton (δ=6.5 ppm) as compared to a DMF internal standard aldehyde proton at (δ=7.8 ppm). Polymerization aliquots taken at t= 0, 0.5, 1.0, 2.0, 3.0, and 6.0 hours.

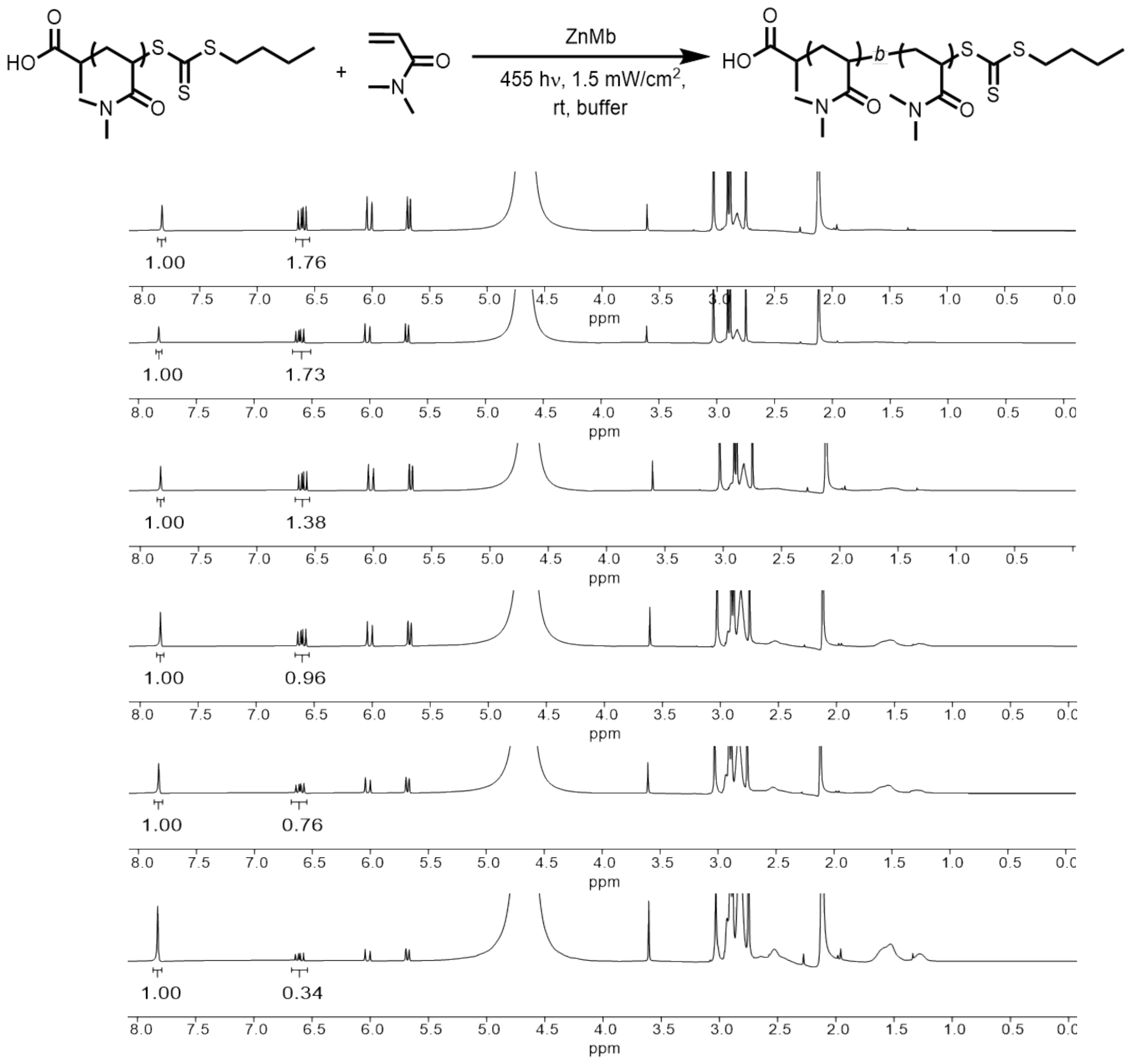

Trial 1

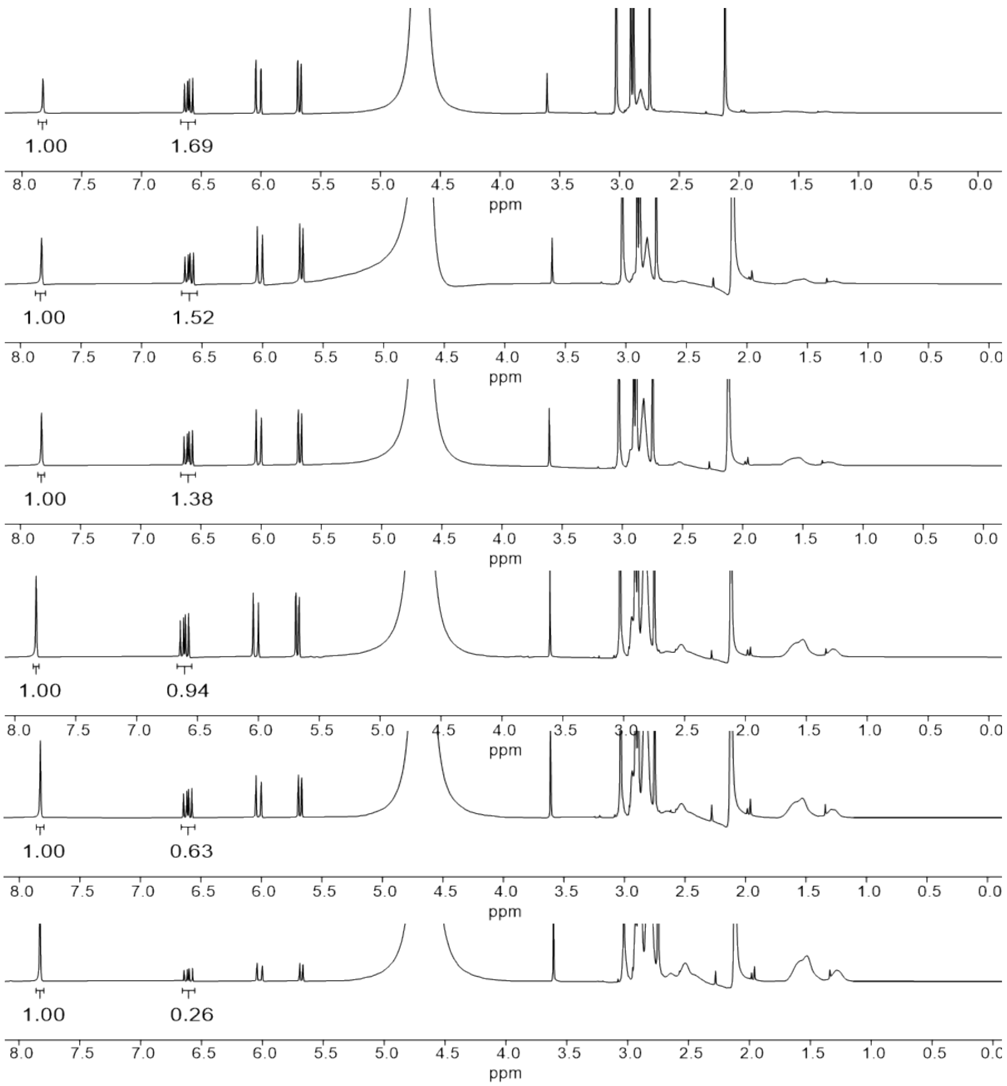
1.00
1.69
8.0 7.5 7.0 6.5 6.0 5.5 5.0 4.5 4.0 3.5 3.0 2.5 2.0 1.5 1.0 0.5 0.0
ppm
1.00
1.52
8.0 7.5 7.0 6.5 6.0 5.5 5.0 4.5 4.0 3.5 3.0 2.5 2.0 1.5 1.0 0.5 0.0
ppm
1.00
1.38
8.0 7.5 7.0 6.5 6.0 5.5 5.0 4.5 4.0 3.5 3.0 2.5 2.0 1.5 1.0 0.5 0.0
ppm
1.00
0.94
8.0 7.5 7.0 6.5 6.0 5.5 5.0 4.5 4.0 3.5 3.0 2.5 2.0 1.5 1.0 0.5 0.0
ppm
1.00
0.63
8.0 7.5 7.0 6.5 6.0 5.5 5.0 4.5 4.0 3.5 3.0 2.5 2.0 1.5 1.0 0.5 0.0
ppm
1.00
0.26
8.0 7.5 7.0 6.5 6.0 5.5 5.0 4.5 4.0 3.5 3.0 2.5 2.0 1.5 1.0 0.5
ppm

Trial 2

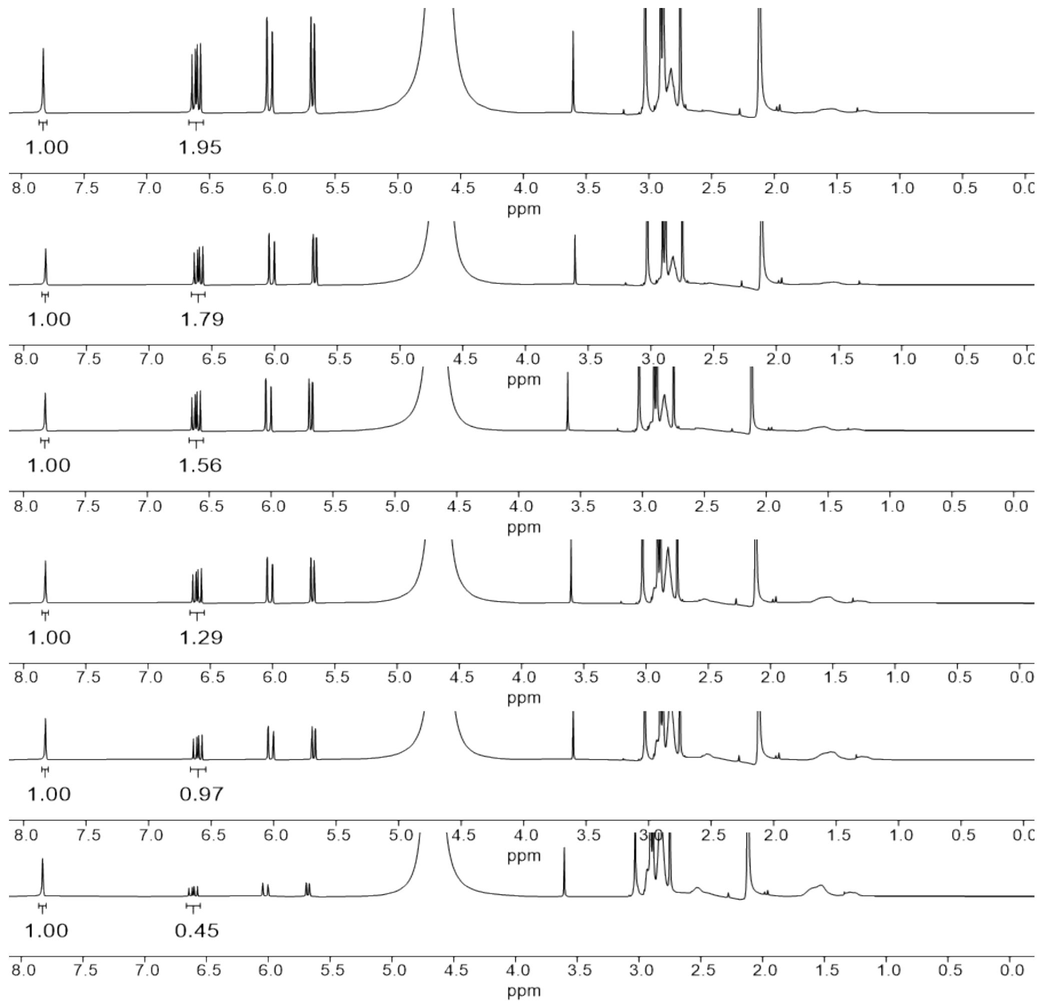

Trial 3

Figure S5. ^{1}H NMR spectra of kinetics for the replicates for PET-RAFT chain extension polymerization of a 22 kg/mol PDMA macro-CTA using zinc myoglobin in tris (0.15 M NaCl) monitored by the disappearance of a DMA vinyl proton (δ=6.5 ppm) as compared to a DMF internal standard aldehyde proton at (δ=7.8 ppm). Polymerization aliquots taken at t= 0, 0.5, 1.0, 2.0, 3.0, and 6.0 hours.

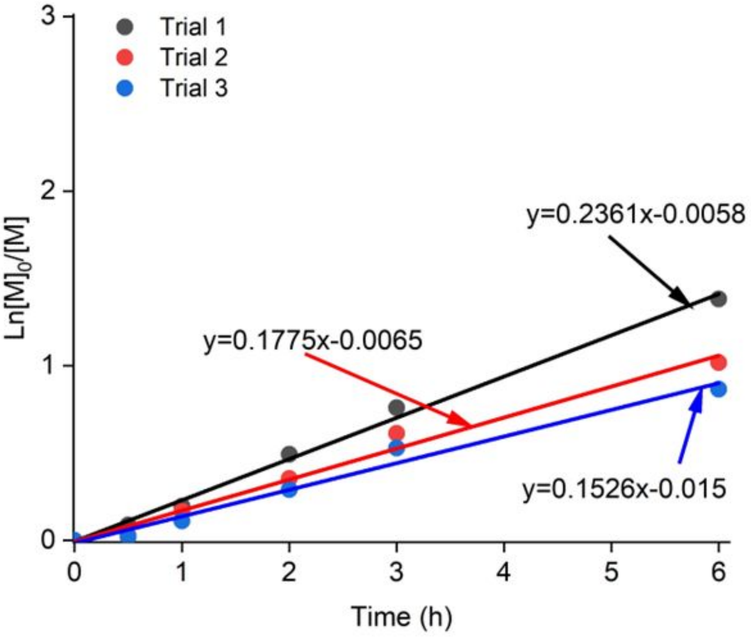

Figure S6. Kinetic plots used to calculate apparent rate constants for chain extension polymerizations using a 22 kg/mol macro-CTA in PBS (0.14 M).

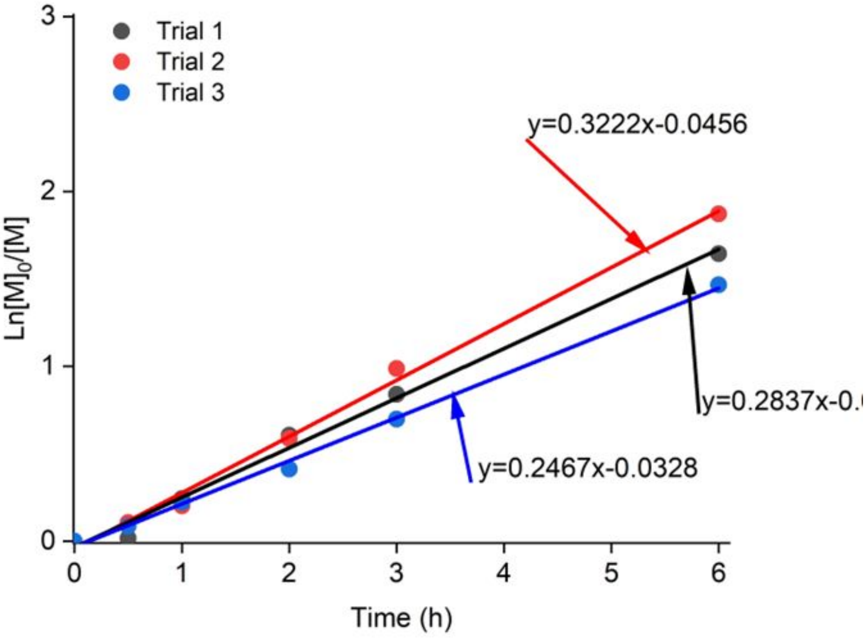

Figure S7. Kinetic plots used to calculate apparent rate constants for chain extension polymerizations using a 22 kg/mol macro-CTA in tris (0.15 M).

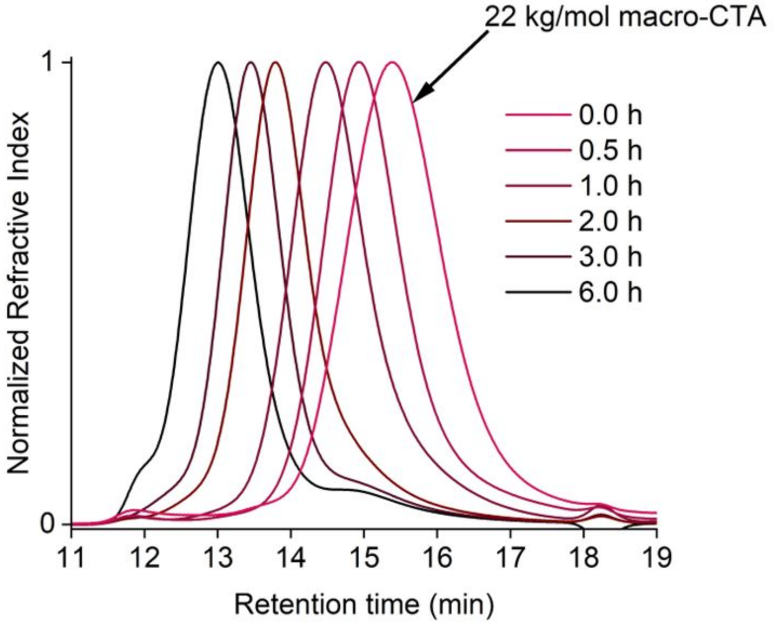

Trial 1

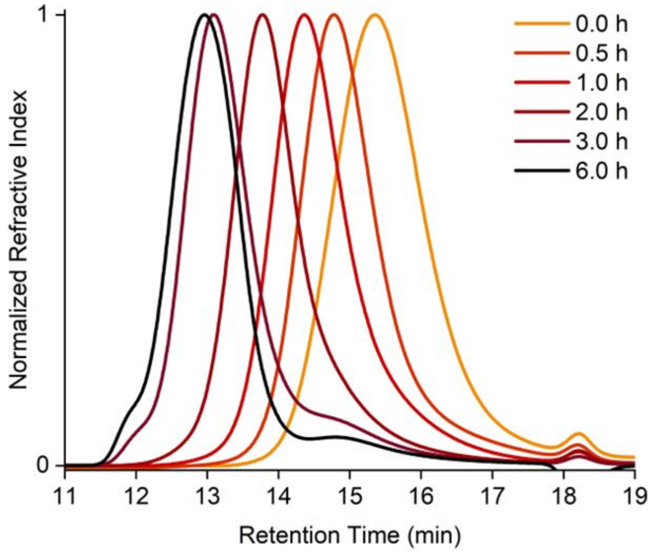

Trial 2

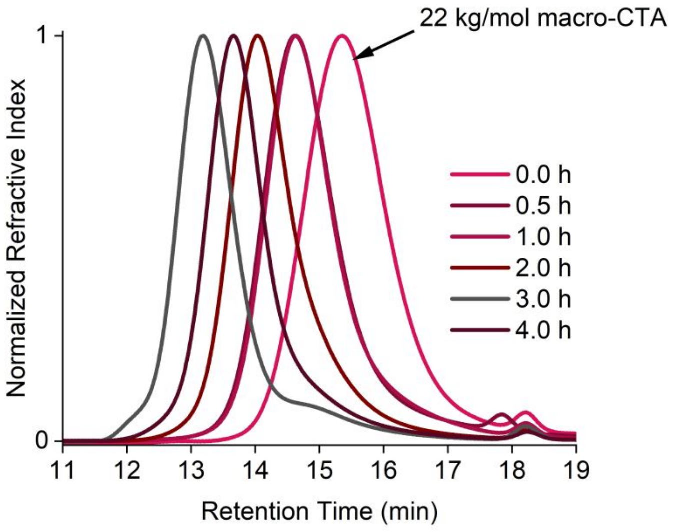

Trial 3

Figure S8. Normalized refractive index traces of the replicates for kinetic experiments for the PET-RAFT chain extension polymerization of a 22 kg/mol PDMA macro-CTA using zinc myoglobin in PBS (0.14 M NaCl).

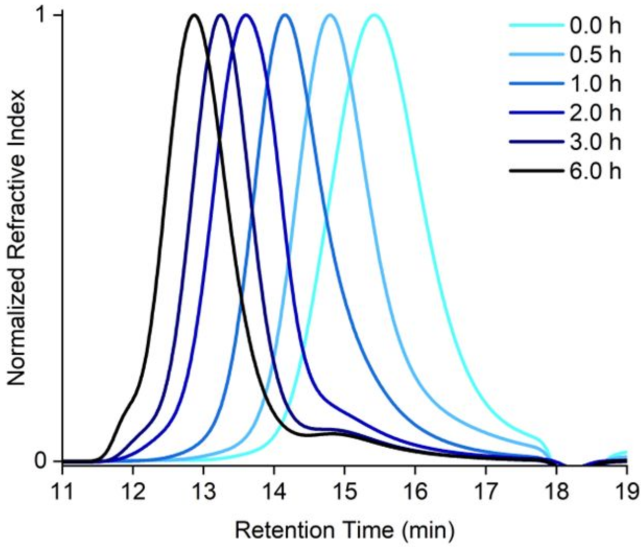

Trial 1

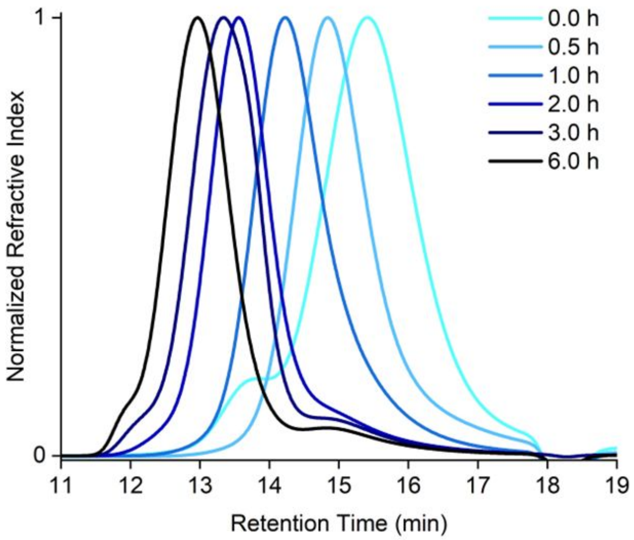

Trial 2

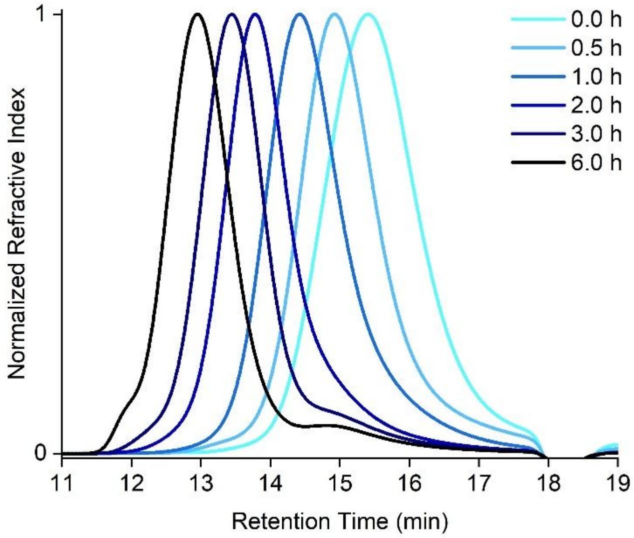

Trial 3

Figure S9. Normalized refractive index traces of the replicates for kinetic experiments for the PET-RAFT chain extension polymerization of a 22 kg/mol PDMA macro-CTA using zinc myoglobin in tris (0.15 M NaCl).

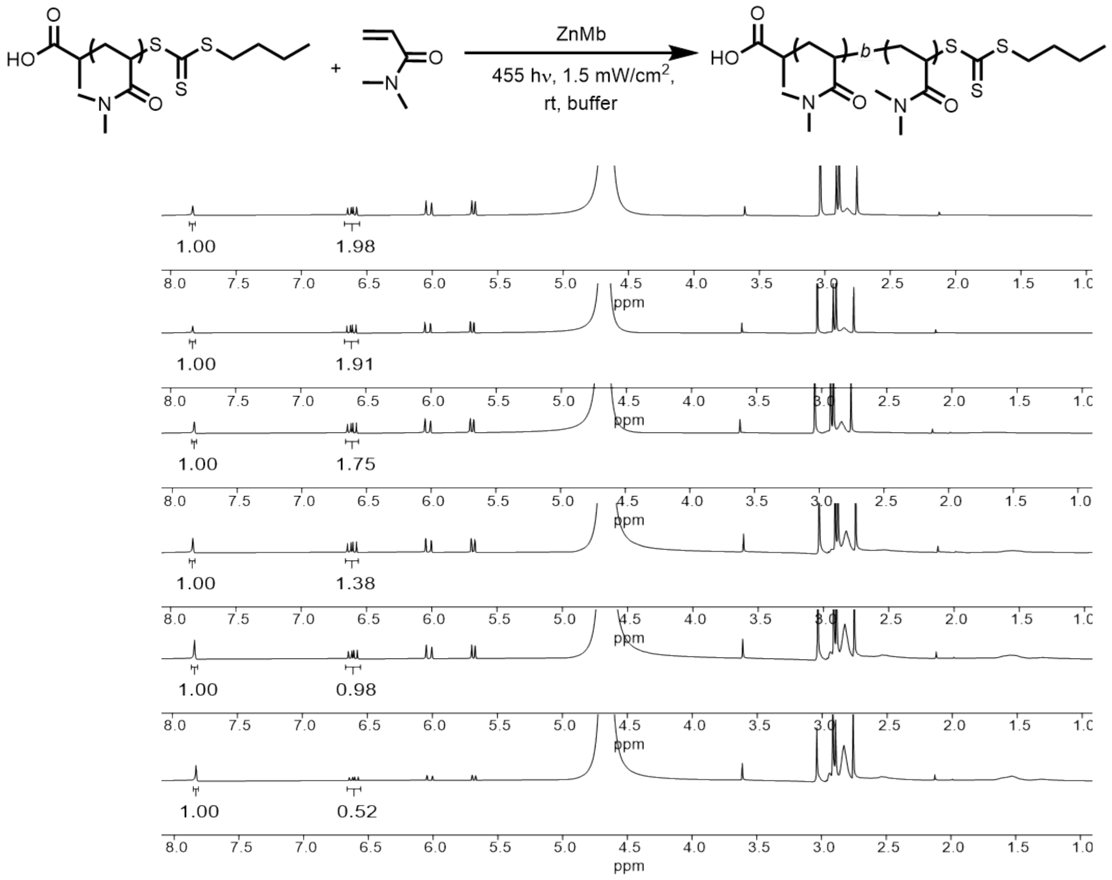
ZnMb
455 hν, 1.5 mW/cm2,
rt, buffer
1.00
1.98
1.00
1.91
1.00
1.75
1.00
1.38
1.00
0.98
1.00
0.52
ppm

Trial 1

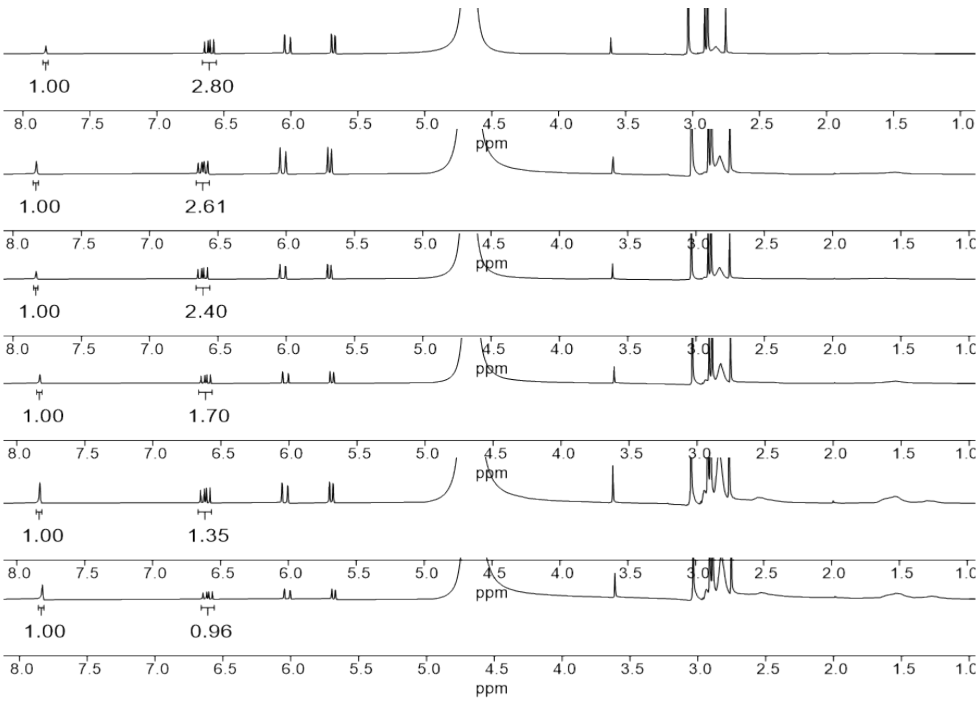

Trial 2

Figure S10. ^{1}H NMR spectra of kinetics for the replicates for PET-RAFT chain extension polymerization of a 22 kg/mol PDMA macro-CTA using zinc myoglobin in tris (0.30 M NaCl) monitored by the disappearance of a DMA vinyl proton (δ=6.5 ppm) as compared to a DMF internal standard aldehyde proton at (δ=7.8 ppm). Polymerization aliquots taken at t= 0, 0.5, 1.0, 2.0, 3.0, and 6.0 hours.

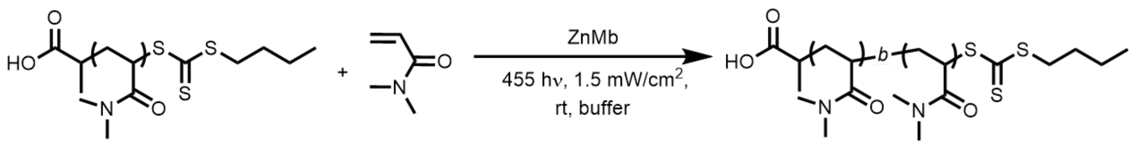

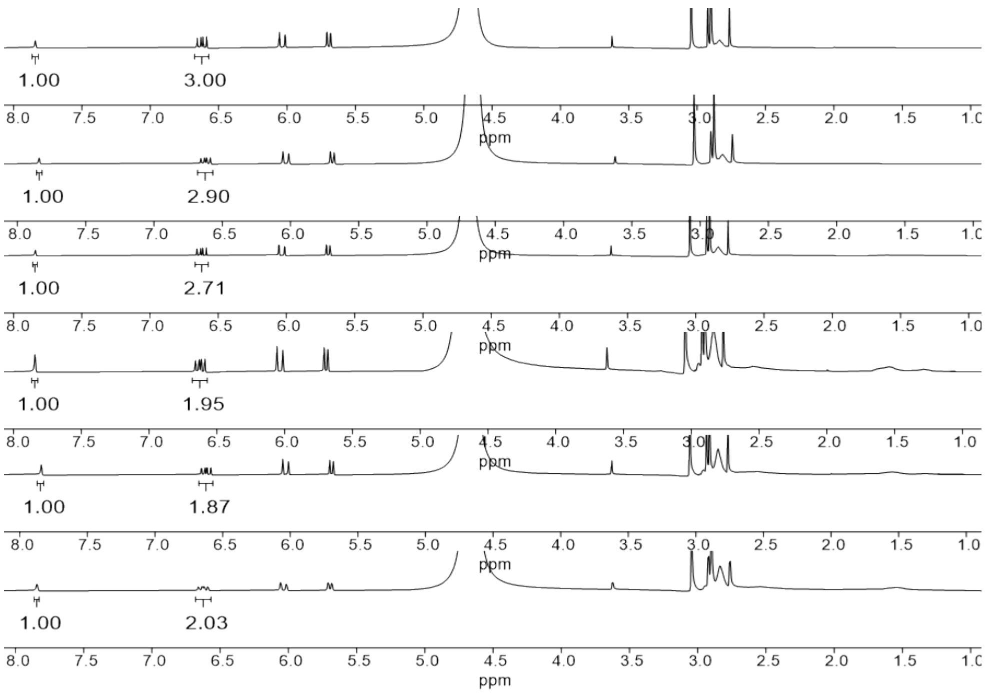

Trial 1

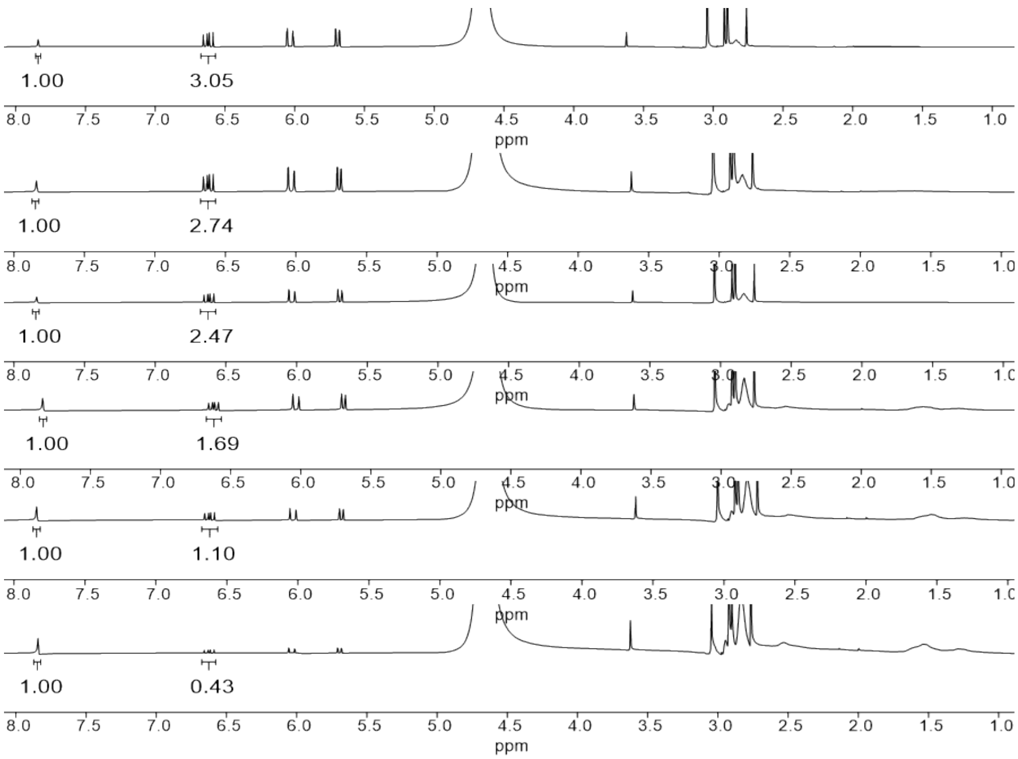

Trial 2

Figure S11. ^{1}H NMR spectra of kinetics for the PET-RAFT chain extension polymerization of a 22 kg/mol PDMA macro-CTA using zinc myoglobin in tris (0.75 M NaCl) monitored by the disappearance of a DMA vinyl proton (δ=6.5 ppm) as compared to a DMF internal standard aldehyde proton at (δ=7.8 ppm). Polymerization aliquots taken at t= 0, 0.5, 1.0, 2.0, 3.0, and 6.0 hours.

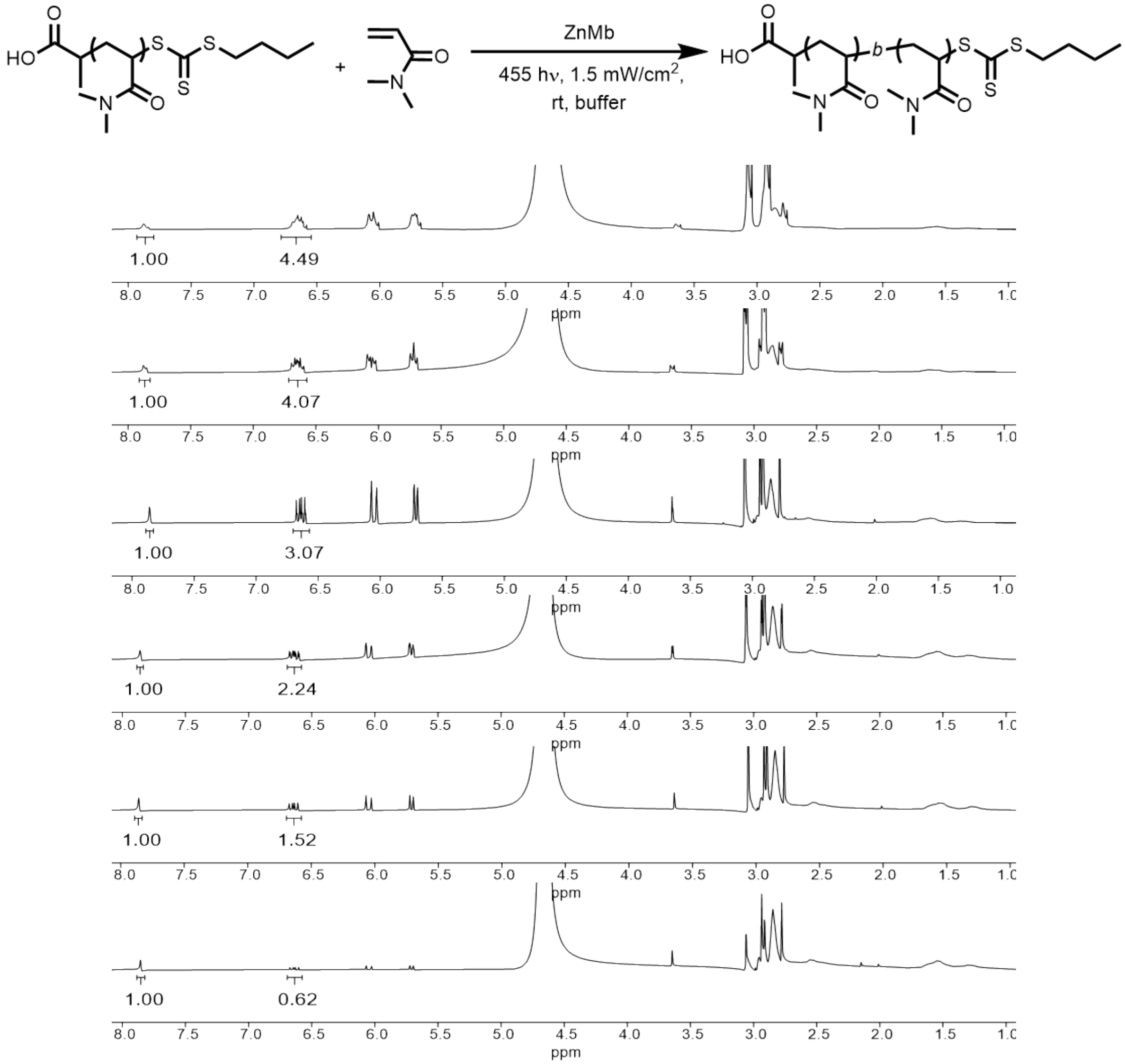
HO
O
S
S
S
N
O
+
N
O
ZnMb
455 hν, 1.5 mW/cm2,
rt, buffer
b
1.00
4.49
1.00
4.07
1.00
3.07
1.00
2.24
1.00
1.52
1.00
0.62
ppm

Trial 1

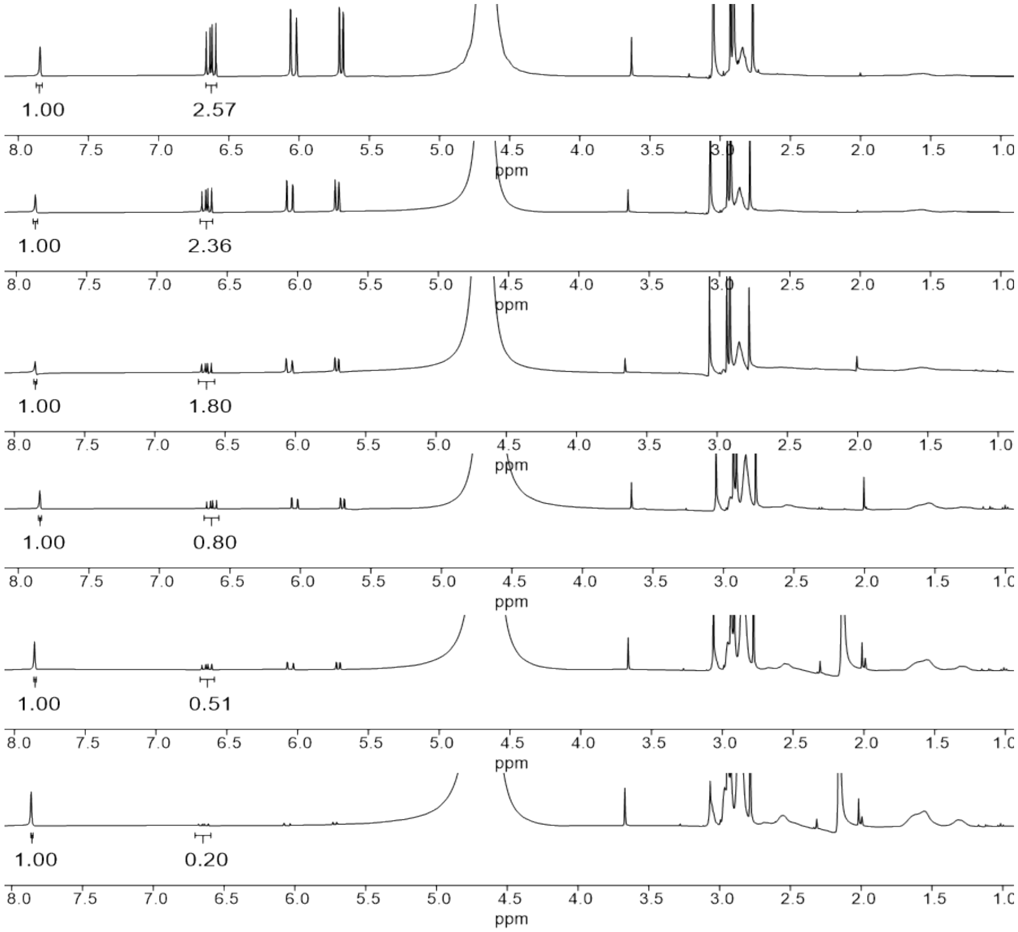

Trial 2

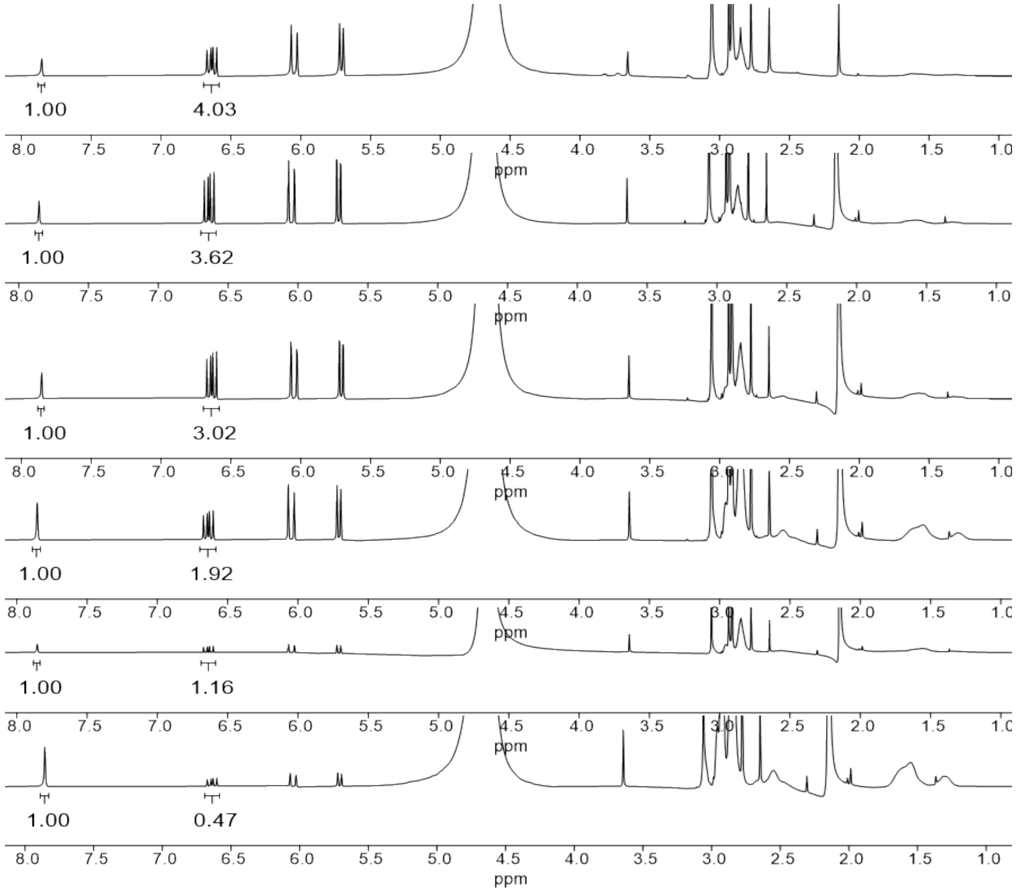

Trial 3

Figure S12. ^{1}H NMR spectra of kinetics for the replicates for PET-RAFT chain extension polymerization of a 22 kg/mol PDMA macro-CTA using zinc myoglobin in tris (1.5 M NaCl) monitored by the disappearance of a DMA vinyl proton (δ=6.5 ppm) as compared to a DMF internal standard aldehyde proton at (δ=7.8 ppm). Polymerization aliquots taken at t= 0, 0.5, 1.0, 2.0, 3.0, and 6.0 hours.

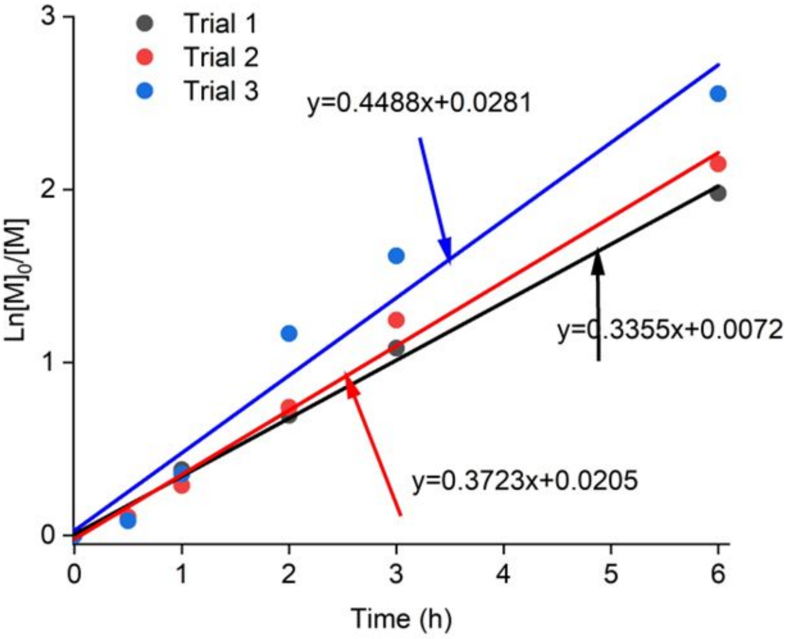

Figure S13. Kinetic plots used to calculate apparent rate constants for chain extension polymerizations using a 22 kg/mol macro-CTA in tris (1.5 M).

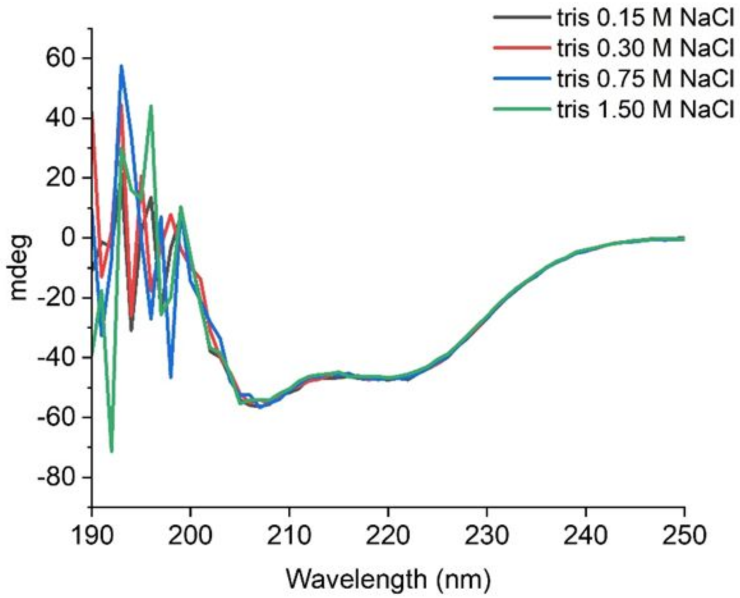

Figure S14. Circular dichroism of zinc myoglobin in tris with different NaCl concentrations.

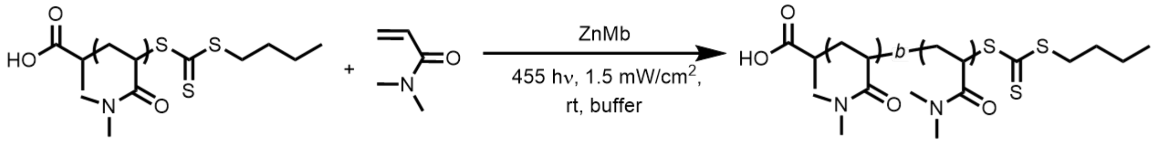

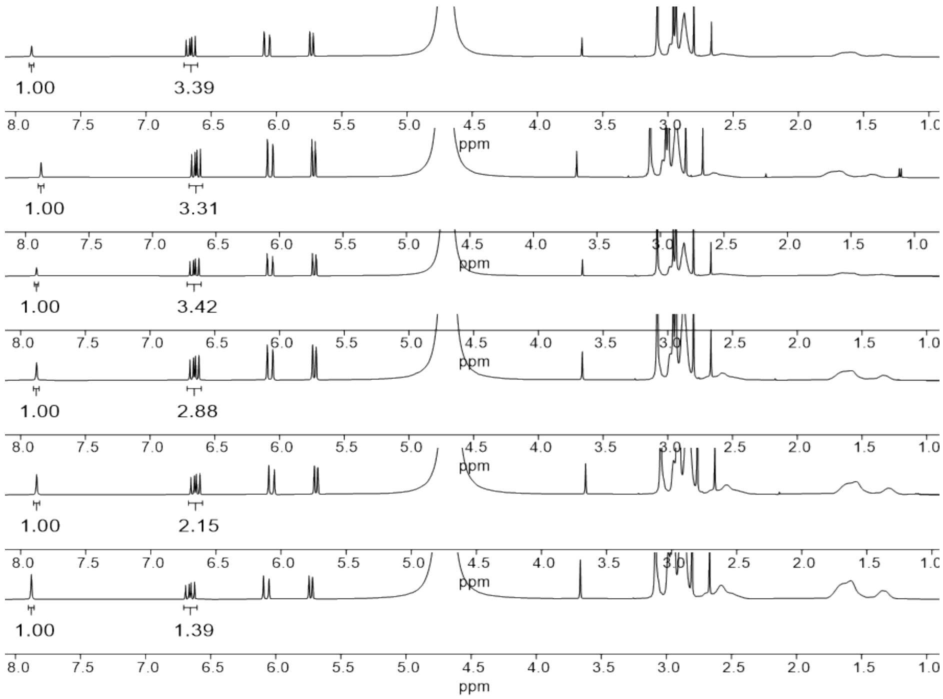

Trial 1

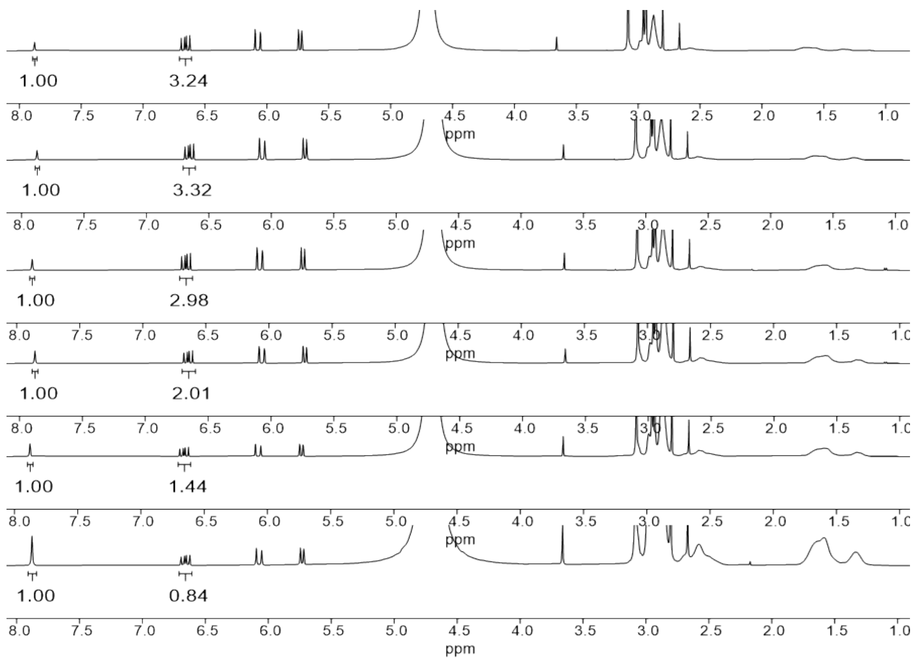

Trial 2

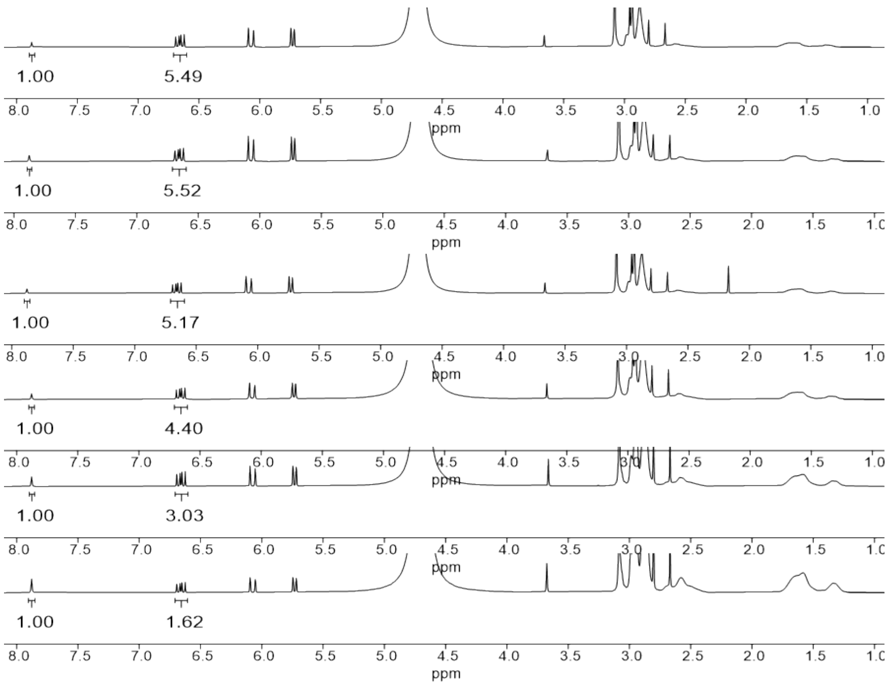

Trial 3

Figure S15. ^{1}H NMR spectra of kinetics for the PET-RAFT chain extension polymerization of a 75 kg/mol PDMA macro-CTA using zinc myoglobin in tris (0.15 M NaCl) monitored by the disappearance of a DMA vinyl proton (δ=6.5 ppm) as compared to a DMF internal standard aldehyde proton at (δ=7.8 ppm). Polymerization aliquots were taken at t= 0, 0.5, 1.0, 2.0, 3.0, and 6.0 hours.

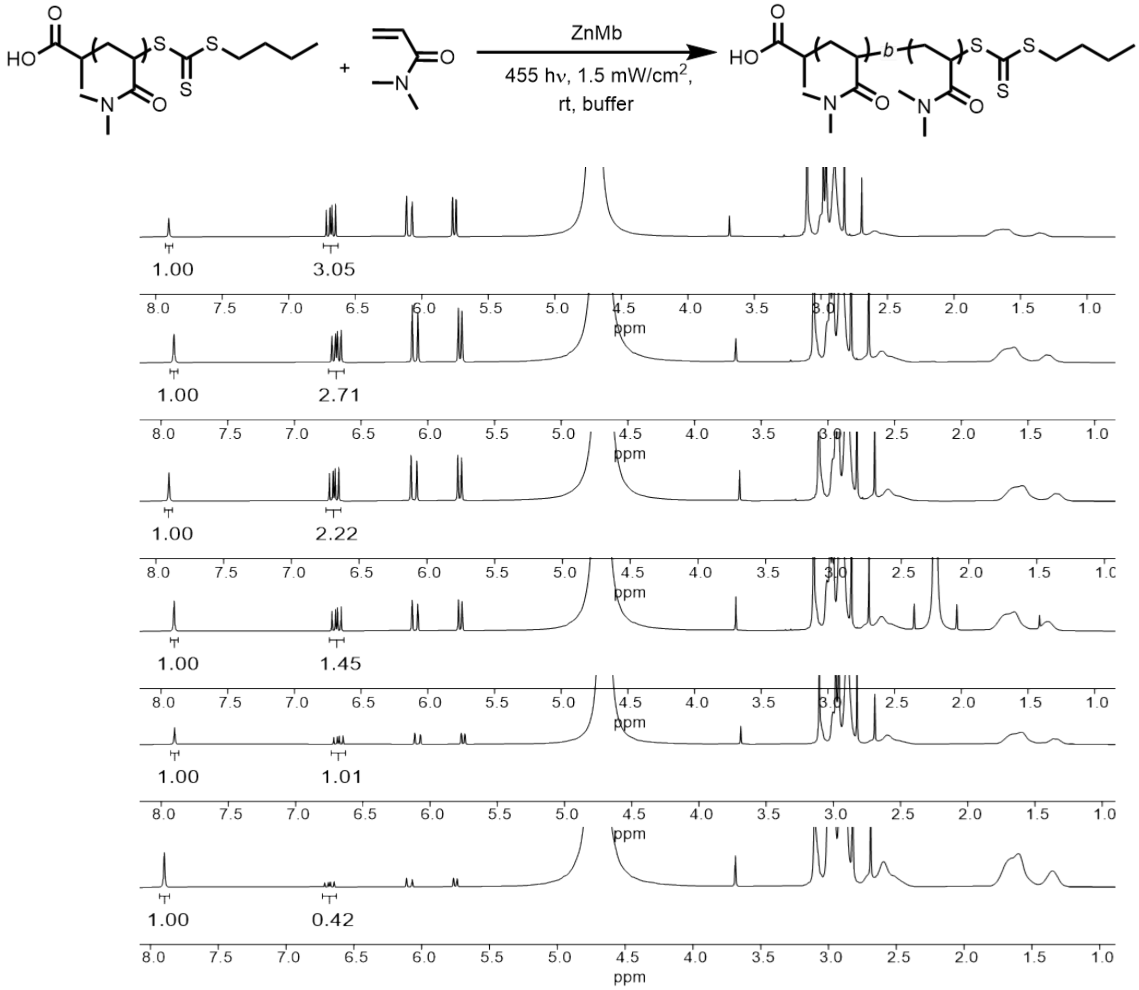

Trial 1

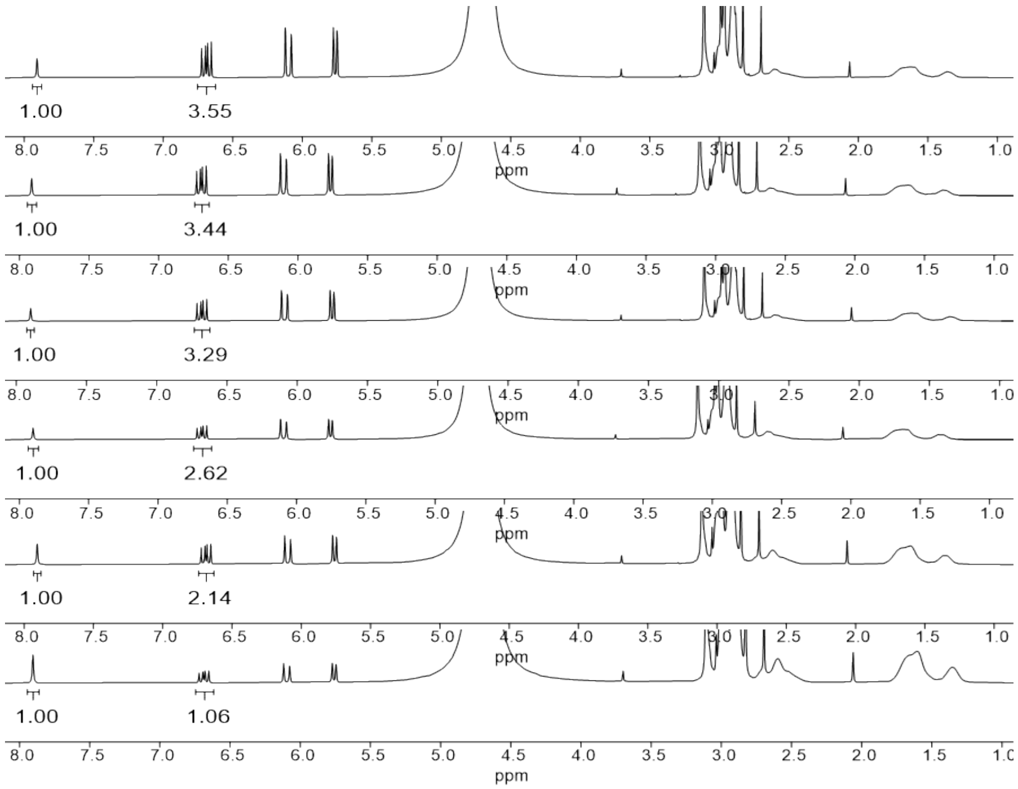
1.00
3.55
1.00
3.44
1.00
3.29
1.00
2.62
1.00
2.14
1.00
1.06
8.0 7.5 7.0 6.5 6.0 5.5 5.0 4.5 4.0 3.5 3.0 2.5 2.0 1.5 1.0
ppm

Trial 2

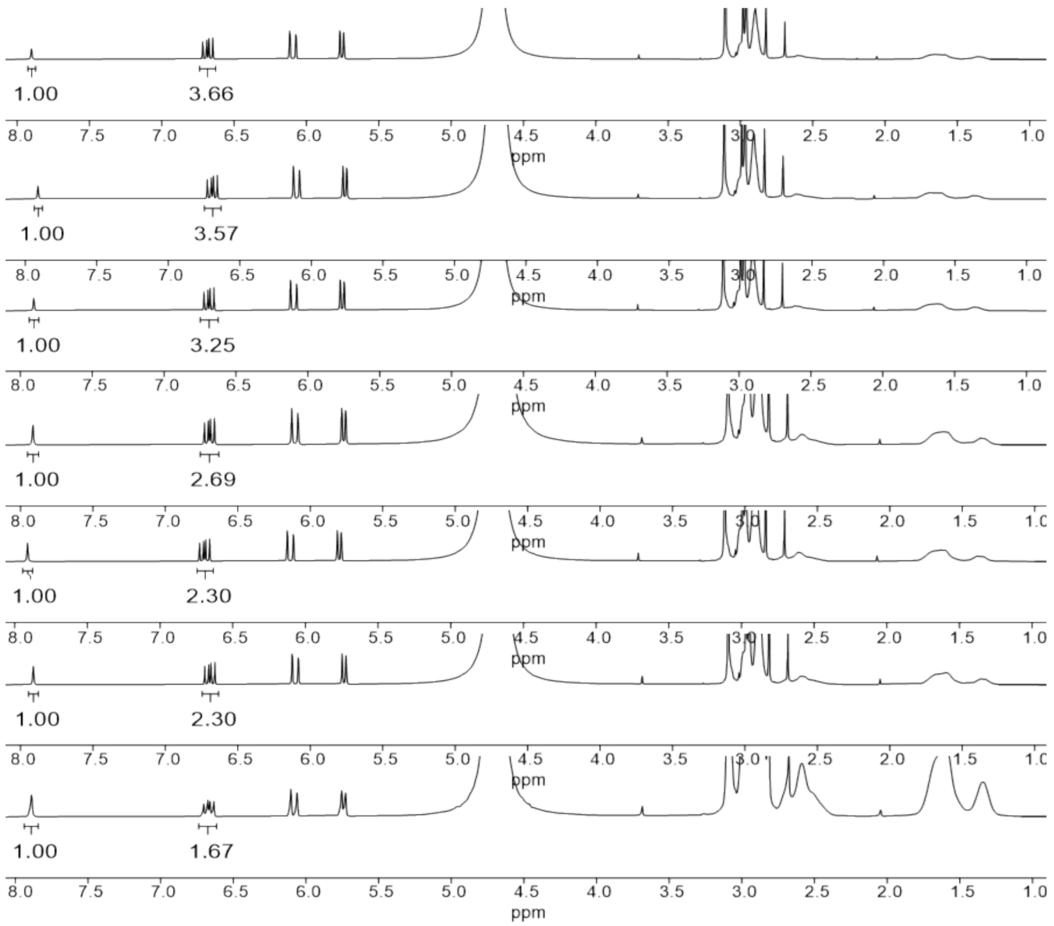

Trial 3

Figure S16. ^{1}H NMR spectra of kinetics for the PET-RAFT chain extension polymerization of a 75 kg/mol PDMA macro-CTA using zinc myoglobin in tris (1.5 M NaCl) monitored by the disappearance of a DMA vinyl proton (δ=6.5 ppm) as compared to a DMF internal standard aldehyde proton at (δ=7.8 ppm). Polymerization aliquots taken at t= 0, 0.5, 1.0, 2.0, 3.0, and 6.0 hours.

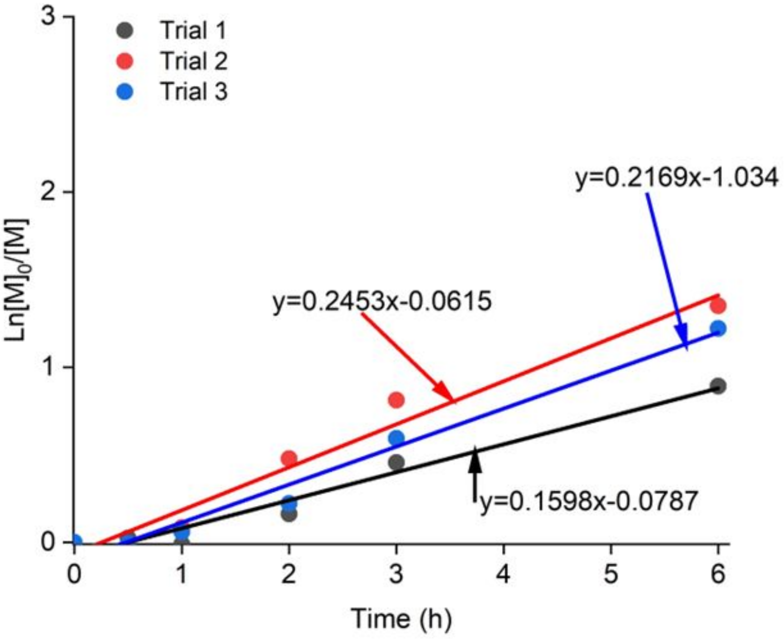

Figure S17. Kinetic plots were used to calculate apparent rate constants for chain extension polymerizations using a 75 kg/mol macro-CTA in tris (0.15 M).

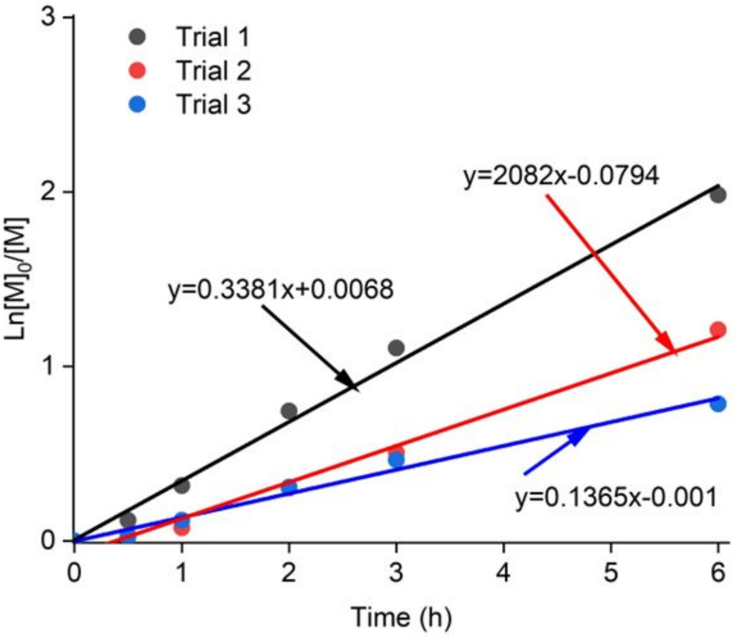

Figure S18. Kinetic plots were used to calculate apparent rate constants for chain extension polymerizations using a 75 kg/mol macro-CTA in tris (1.5 M NaCl).

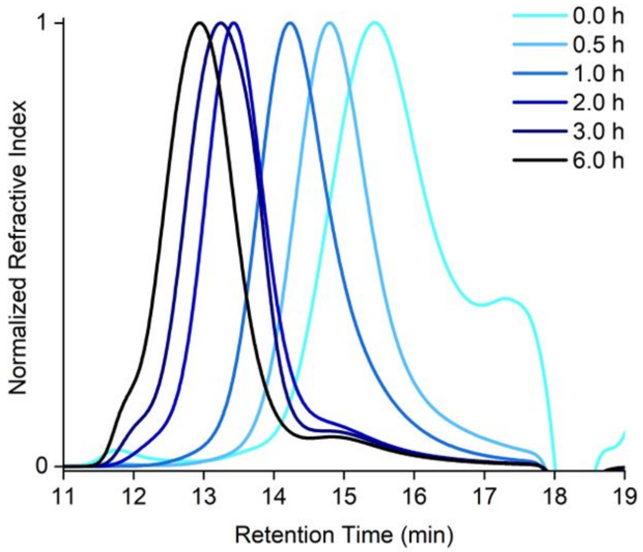

Trial 2

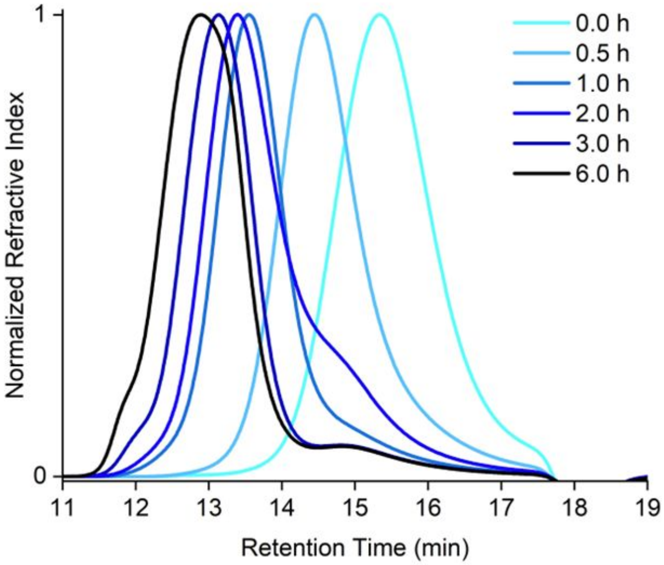

Trial 3

Figure S19. Normalized refractive index traces of the replicates for kinetic experiments for the PET-RAFT chain extension polymerization of a 22 kg/mol PDMA macro-CTA using zinc myoglobin in tris (1.5 M NaCl). Trial 1 can be found in (**Figure 3d**).

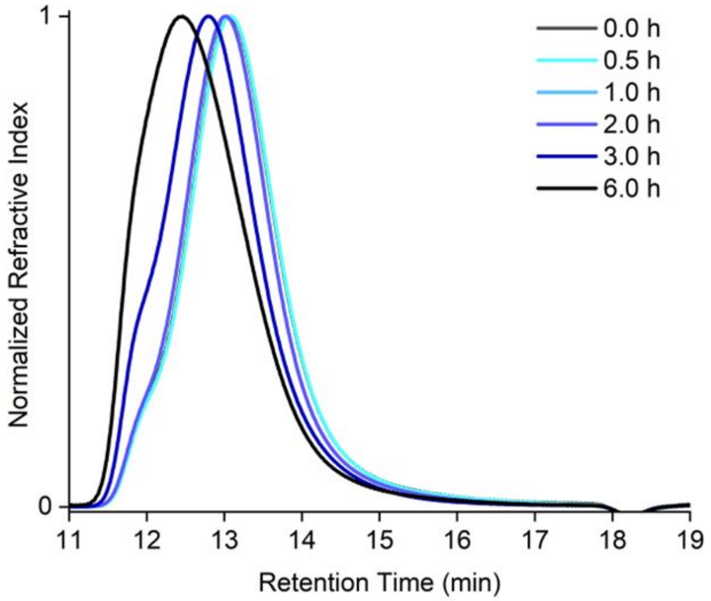

Trial 1

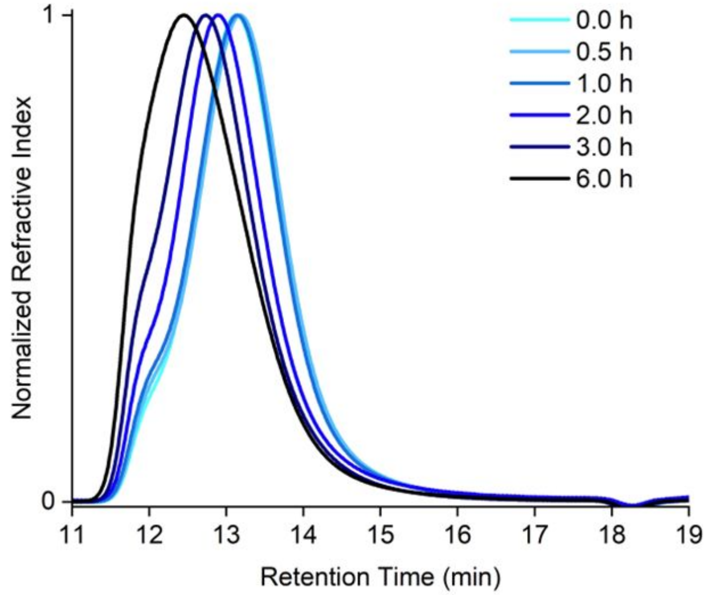

Trial 2

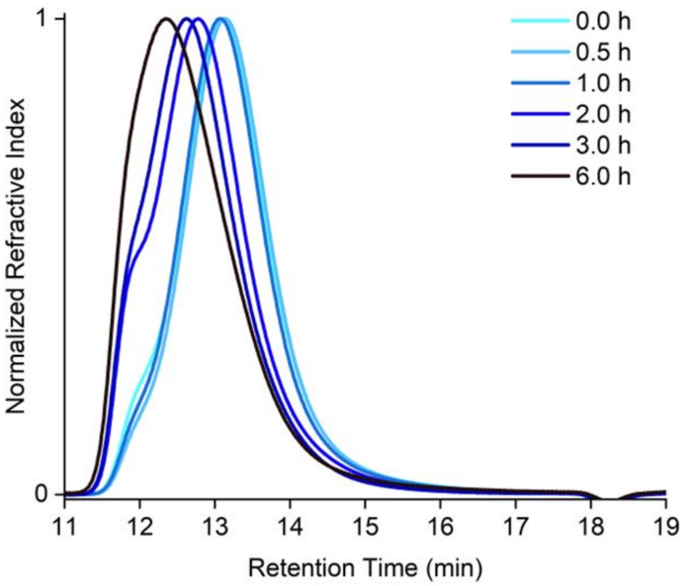

Trial 3

Figure S20. Normalized refractive index traces of kinetic experiments for the PET-RAFT chain extension polymerization of a 75 kg/mol PDMA macro-CTA using zinc myoglobin in tris (0.15 M NaCl).

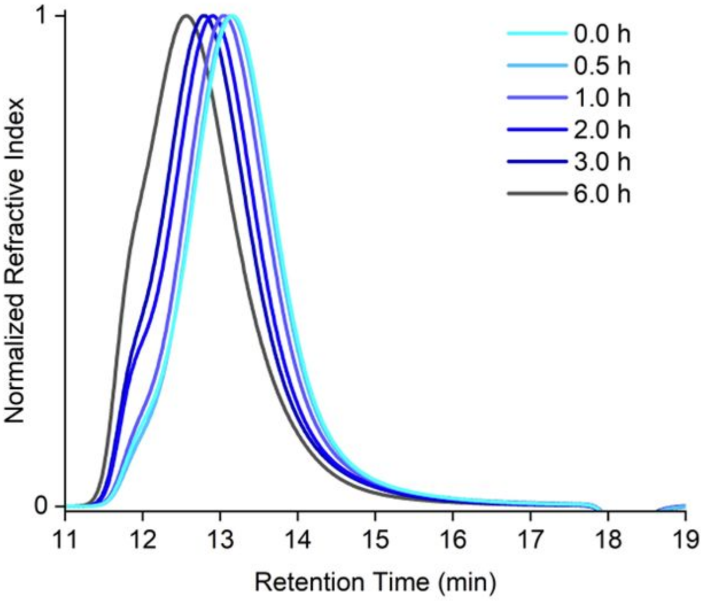

Trial 2

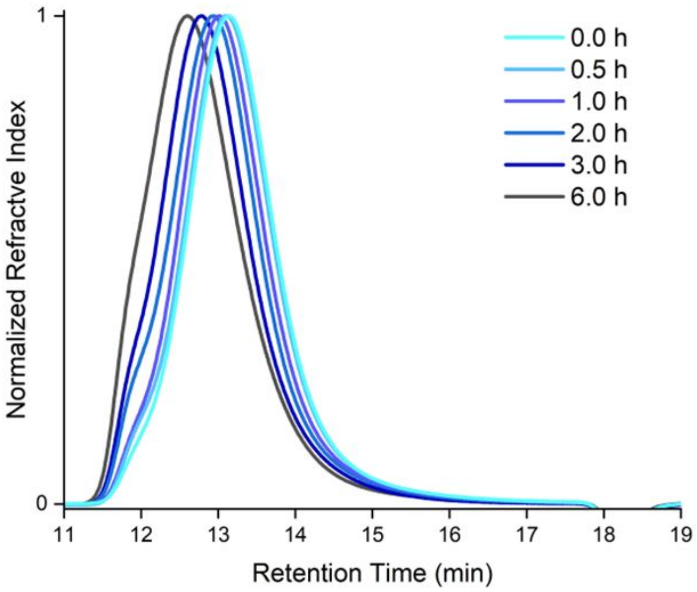

Trial 3

Figure S21. Normalized refractive index traces of kinetic experiments for the PET-RAFT chain extension polymerization of a 75 kg/mol PDMA macro-CTA using zinc myoglobin in tris (1.5 M NaCl). Trial 1 can be found in (**Figure 3e**).

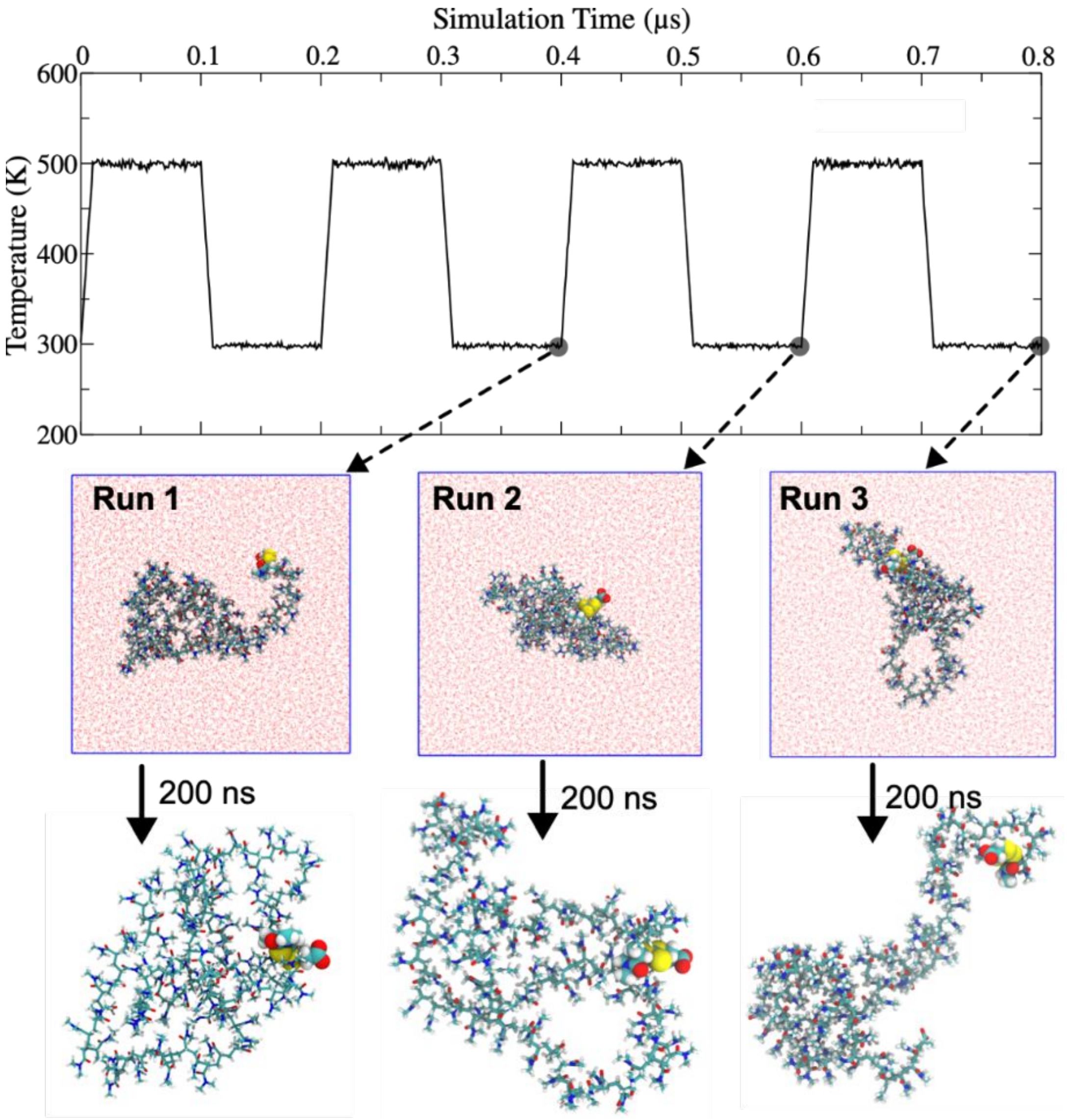

Figure S22. Annealing atomistic simulation to relax the polymer chain for DP = 100. Four annealing cycles were conducted, with 200 ns each. For each annealing cycle, the system temperature was increased from 298K to 500 K within 10 ns, stayed at 500 K for 90 ns, decreased to 298 K within 10 ns, and stayed at 298 K for another 90 ns. Based on the simulation structures at 400 ns, 600 ns, and 800 ns (all at 298 K), three parallel simulations were carried out, each lasting another 200 ns, where the data were collected and analyzed. In the simulation snapshots, the initial and the final structures in the three parallel runs are presented. The polymer tail groups are highlighted with the S/O/N/C/H atoms colored in yellow/red/blue/cyan/white, respectively.

Supporting Tables:

		0.0 h	0.5 h	1.0 h	2.0 h	3.0 h	6.0 h
Trial 1	M_w (g/mol)<br>M_n (g/mol)<br>*Đ*	24,300<br>23,000<br>1.06	29,000<br>27,000<br>1.07	36,900<br>33,100<br>1.11	54,700<br>48,400<br>1.13	70,400<br>6100<br>1.15	93,900<br>76,700<br>1.22
Trial 2	M_w (g/mol)<br>M_n (g/mol)<br>*Đ*		29,200<br>26,890<br>1.08	37,100<br>33,500<br>1.11	52,300<br>45,600<br>1.15	83,400<br>73,700<br>1.13	104,300<br>102,000<br>1.04
Trial 3	M_w (g/mol)<br>M_n (g/mol)<br>*Đ*	23,700<br>23,000<br>1.03	27,700<br>26,300<br>1.03	33,300<br>30,610<br>1.09	44,800<br>39,00<br>1.13	57,600<br>49,600<br>1.16	76,800<br>66,000<br>1.16

Table S1. Table containing molecular weight data for timepoints taken during the chain extension polymerization of a 22 kg/mol macro-CTA in PBS (0.14 M NaCl).

		0.0 h	0.5 h	1.0 h	2.0 h	3.0 h	6.0 h
Trial 1	M_w (g/mol)<br>M_n (g/mol)<br>*Đ*	26,200<br>22,400<br>1.15	30,700<br>28,200<br>1.09	42,900<br>38,600<br>1.11	62,800<br>53,800<br>1.17	81,100<br>74,300<br>1.09	103,500<br>93,000<br>1.11
Trial 2	M_w (g/mol)<br>M_n (g/mol)<br>*Đ*	23,800<br>21,200<br>1.12	30,000<br>27,900<br>1.08	40,900<br>36,500<br>1.112	66,200<br>56,600<br>1.1	79,990<br>73,800<br>1.08	101,000<br>94,700<br>1.07
Trial 3	M_w (g/mol)<br>M_n (g/mol)<br>*Đ*	21,900<br>20,900<br>1.05	29,400<br>27,000<br>1.09	37,900<br>34,100<br>1.11	55,300<br>48,300<br>1.1	72,200<br>61,900<br>1.17	99,400<br>88,500<br>1.1

Table S2. Table containing molecular weight data for timepoints taken during the chain extension polymerization of a 22 kg/mol macro-CTA in tris (0.15 M NaCl)

		0.0 h	0.5 h	1.0 h	2.0 h	3.0 h	6.0 h
Trial 1	M_w(g/mol)<br>M_n (g/mol)<br>*Đ*	30,900<br>30,300<br>1.02	32,200<br>30,400<br>1.06	42,400<br>38,200<br>1.11	71,700<br>64,700<br>1.11	82,800<br>73,000<br>1.13	114,000<br>106,000<br>1.078
Trial 2	M_w(g/mol)<br>M_n (g/mol)<br>*Đ*	22,400<br>19,670<br>1.14	32,200<br>30,300<br>1.06	41,900<br>37,800<br>1.11	71,200<br>65,300<br>1.09	87,500<br>81,200<br>1.08	109,000<br>98,900<br>1.10
Trial 3	M_w (g/mol)<br>M_n (g/mol)<br>*Đ*	25,200<br>24,600<br>1.02	36,800<br>33,800<br>1.09	62,300<br>51,400<br>1.21	63,400<br>58,300<br>1.09	90,300<br>83,200<br>1.21	111,000<br>98,900<br>1.12

Table S3. Table containing molecular weight data for timepoints taken during the chain extension polymerization of a 22 kg/mol macro-CTA in tris (1.5 M NaCl).

		0.0 h	0.5 h	1.0 h	2.0 h	3.0 h	6.0 h
Trial 1	M_w (g/mol)<br>M_n (g/mol)<br>*Đ*	91,200<br>79,100<br>1.15	90,900<br>79,300<br>1.1	91,000<br>79,900<br>1.134	96,300<br>85,000<br>1.13	116,000<br>97,300<br>1.19	140,00<br>114,00<br>1.22
Trial 2	M_w (g/mol)<br>M_n (g/mol)<br>*Đ*	90,700<br>78,300<br>1.16	87,200<br>76,800<br>1.14	90,900<br>80,400<br>1.13	122,000<br>101,000<br>1.21	133,000<br>111,000<br>1.20	154,000<br>125,000<br>1.23
Trial 3	M_w (g/mol)<br>M_n (g/mol)<br>*Đ*	90,100<br>77,900<br>1.16	91,300<br>79,300<br>1.15	94,200<br>81,000<br>1.16	108,000<br>91,300<br>1.1	122,000<br>103,000<br>1.19	146,000<br>119,000<br>1.23

Table S4. Table containing molecular weight data for timepoints taken during the chain extension polymerization of a 75 kg/mol macro-CTA in tris (0.15 M NaCl).

		0.0 h	0.5 h	1.0 h	2.0 h	3.0 h	6.0 h
Trial 1	M_w (g/mol)<br>M_n (g/mol)<br>*Đ*	81,900<br>72,500<br>1.13	90,200<br>84,300<br>1.14	98,100<br>85,900<br>1.14	119,000<br>104,000<br>1.15	131,000<br>114,000<br>1.14	145,000<br>134,000<br>1.08
Trial 2	M_w (g/mol)<br>M_n (g/mol)<br>*Đ*	82,400<br>73,500<br>1.12	83,000<br>75,500<br>1.10	88,100<br>78,400<br>1.12	87,900<br>100,000<br>1.14	108,000<br>95,400<br>1.14	129,000<br>111,000<br>1.1
Trial 3	M_w (g/mol)<br>M_n (g/mol)<br>*Đ*	84,900<br>75,700<br>1.12	88,600<br>78,000<br>1.13	92,600<br>82,000<br>1.13	105,000<br>91,900<br>1.15	113,000<br>98,000<br>1.15	132,00<br>113,000<br>1.16

Table S5. Table containing molecular weight data for timepoints taken during the chain extension polymerization of a 75 kg/mol macro-CTA in tris (1.5 M NaCl).

SASA with a protein-sized probe (radius 1.75 nm)						
		**DP = 50**	**100**	**200**	**400**	**600**
Tail group	Run 1 [a]	7.1 ± 4.1	8.2 ± 9.4	3.8 ± 4.7	2.7 ± 3.1	0.1 ± 0.2
	Run 2 [a]	6.6 ± 4.6	12.4 ± 8.8	6.3 ± 5.5	0.2 ± 0.6	4.6 ± 2.2
	Run 3 [a]	11.6 ± 6.7	10.7 ± 6.8	1.4 ± 1.4	2.0 ± 2.0	3.5 ± 2.6
	**AVE.** [b]	**8.4 ± 2.8**	**10.4 ± 2.1**	**3.8 ± 2.4**	**1.6 ± 1.3**	**2.7 ± 2.3**
Polymer [c]	Run 1 [a]	2.9 ± 0.1	2.0 ± 0.2	1.13 ± 0.03	0.89 ± 0.03	0.64 ± 0.01
	Run 2 [a]	2.9 ± 0.2	1.9 ± 0.1	1.23 ± 0.04	0.83 ± 0.04	0.62 ± 0.01
	Run 3 [a]	2.9 ± 0.1	1.9 ± 0.1	1.23 ± 0.03	0.8 ± 0.02	0.79 ± 0.02
	**AVE.** [b]	**2.9 ± 0.0**	**2.0 ± 0.1**	**1.2 ± 0.1**	**0.84 ± 0.04**	**0.68 ± 0.09**
**SASA with a water-sized probe** (radius 0.14 nm)						
		**DP = 50**	**100**	**200**	**400**	**600**
Tail group	Run 1 [a]	2.0 ± 0.4	2.3 ± 0.8	1.6 ± 0.6	2.1 ± 0.5	1.2 ± 0.2
	Run 2 [a]	2.0 ± 0.5	2.9 ± 0.7	2.0 ± 0.8	1.3 ± 0.4	1.9 ± 0.4
	Run 3 [a]	2.5 ± 0.7	2.7 ± 0.6	1.0 ± 0.2	1.5 ± 0.4	2.0 ± 0.5
	**AVE.** [b]	**2.2 ± 0.3**	**2.6 ± 0.3**	**1.5 ± 0.5**	**1.6 ± 0.4**	**1.7 ± 0.4**
Polymer [c]	Run 1 [a]	0.92 ± 0.06	0.83 ± 0.05	0.65 ± 0.03	0.64 ± 0.02	0.57 ± 0.02
	Run 2 [a]	0.93 ± 0.05	0.79 ± 0.04	0.72 ± 0.03	0.63 ± 0.03	0.57 ± 0.01
	Run 3 [a]	0.90 ± 0.06	0.80 ± 0.05	0.67 ± 0.03	0.60 ± 0.02	0.60 ± 0.01
	**AVE.** [b]	**0.92 ± 0.02**	**0.81 ± 0.02**	**0.68 ± 0.04**	**0.62 ± 0.02**	**0.58 ± 0.02**

Table S6. Solvent accessible surface area (nm^2). Table containing (a) the ensemble average of the SASA of each simulation run of 200 ns. Error bars are the standard deviation. (b) The block average of three parallel runs. The error bars are for the standard deviation. (c) The total SASA of the whole polymer chain divided by the polymer DP.

References:

(1) Cowan, J. A.; Gray, H. B. Synthesis and properties of metal-substituted myoglobins. *Inorg. Chem.* **1989**, *28*, 2074-2078.

(2) Adler, A. D.; Longo, F. R.; Kampas, F.; Kim, J. On the preparation of metalloporphyrins. *J. Inorg. Nucl. Chem.* **1970**, *32*, 2443-2445.

(3) Skey, J.; O'Reilly, R. K. Facile one pot synthesis of a range of reversible addition–fragmentation chain transfer (RAFT) agents. *Chem. Commun.* **2008**, 4183-4185.

(4) Hess, B.; Kutzner, C.; van der Spoel, D.; Lindahl, E. GROMACS 4: Algorithms for Highly Efficient, Load Balanced, and Scalable Molecular Simulation. *J. Chem. Theory Comput.* **2008**, *4*, 435-447.

(5) Vanommeslaeghe, K.; Raman, E. P.; MacKerell, A. D. Automation of the CHARMM General Force Field (CGenFF) II: Assignment of Bonded Parameters and Partial Atomic Charges. *J. Chem. Inf. Model.* **2012**, *52*, 3155-3168.

(6) Vanommeslaeghe, K.; MacKerell, A. D. Automation of the CHARMM General Force Field (CGenFF) I: Bond Perception and Atom Typing. *J. Chem. Inf. Model.* **2012**, *52*, 3144-3154.

(7) Lee, J.; Cheng, X.; Swails, J. M.; Yeom, M. S.; Eastman, P. K.; Lemkul, J. A.; Wei, S.; Buckner, J.; Jeong, J. C.; Qi, Y.; Jo, S.; Pande, V. S.; Case, D. A.; Brooks, C. L.; MacKerell, A. D.; Klauda, J. B.; Im, W. CHARMM-GUI Input Generator for NAMD, GROMACS, AMBER, OpenMM, and CHARMM/OpenMM Simulations Using the CHARMM36 Additive Force Field. *J. Chem. Theory Comput.* **2016**, *12*, 405-413.

(8) MacKerell, A. D.; Bashford, D.; Bellott, M.; Dunbrack, R. L.; Evanseck, J. D.; Field, M. J.; Fischer, S.; Gao, J.; Guo, H.; Ha, S.; Joseph-McCarthy, D.; Kuchnir, L.; Kuczera, K.; Lau, F. T. K.; Mattos, C.; Michnick, S.; Ngo, T.; Nguyen, D. T.; Prodhom, B.; Reiher, W. E.; et al. All-Atom Empirical Potential for Molecular Modeling and Dynamics Studies of Proteins. *J. Phys. Chem. B* **1998**, *102*, 3586-3616.

(9) Miyamoto, S.; Kollman, P. A. SETTLE: An Analytical Version of the SHAKE and RATTLE Algorithm for Rigid Water Models. *J. Comput. Chem.* **1992**, *13*, 952-962.

(10) Sun, H.; Qiao, B.; Choi, W.; Hampu, N.; McCallum, N. C.; Thompson, M. P.; Oktawiec, J.; Weigand, S.; Ebrahim, O. M.; Olvera de la Cruz, M.; Gianneschi, N. C. Origin of Proteolytic Stability of Peptide-Brush Polymers as Globular Proteomimetics. *ACS Cent. Sci.* **2021**, *7*, 2063-2072.

(11) Panganiban, B.; Qiao, B.; Jiang, T.; DelRe, C.; Obadia, M. M.; Nguyen, T. D.; Smith, A. A. A.; Hall, A.; Sit, I.; Crosby, M. G.; Dennis, P. B.; Drockenmuller, E.; Olvera de la Cruz, M.; Xu, T. Random Heteropolymers Preserve Protein Function in Foreign Environments. *Science* **2018**, *359*, 1239-1243.

(12) Qiao, B.; Jiménez-Ángeles, F.; Nguyen, T. D.; Olvera de la Cruz, M. Water follows polar and nonpolar protein surface domains. *Proc. Natl. Acad. Sci. U. S. A.* **2019**, *116*, 19274-19281.

(13) Darden, T.; York, D.; Pedersen, L. Particle Mesh Ewald: An N·log(N) Method for Ewald Sums in Large Systems. *J. Chem. Phys.* **1993**, *98*, 10089-10092.

(14) Essmann, U.; Perera, L.; Berkowitz, M. L.; Darden, T.; Lee, H.; Pedersen, L. A Smooth Particle Mesh Ewald Method. *J. Chem. Phys.* **1995**, *103*, 8577-8593.

(15) Hess, B. P-LINCS: A Parallel Linear Constraint Solver for Molecular Simulation. *J. Chem. Theory Comput.* **2008**, *4*, 116-122.

(16) Hess, B.; Bekker, H.; Berendsen, H. J. C.; Fraaije, J. G. E. M. LINCS: A Linear Constraint Solver for Molecular Simulations. *J. Comput. Chem.* **1997**, *18*, 1463-1472.

(17) Wang, M.; Choi, M.; Battistella, C.; Gattis, B.; Qiao, B.; Evangelopoulos, M.; Mirkin, C.; Olvera de la Cruz, M.; Zhang, B.; Gianneschi, N. C. Proteomimetic Polymers Trigger Potent Antigen-Specific T Cell Responses to Limit Tumor Growth. *J. Am. Chem. Soc.* **2024**, *146*, 14959–14971.

(18) Oktawiec, J.; Ebrahim, O. M.; Chen, Y.; Su, K.; Sharpe, C.; Rosenmann, N. D.; Barbut, C.; Weigand, S. J.; Thompson, M. P.; Byrnes, J.; Qiao, B.; Gianneschi, N. C. Conformational modulation and polymerization-induced folding of proteomimetic peptide brush polymers. *Chem. Sci.* **2024**, *15*, 13899-13908.

(19) Du, F.; Rische, C. H.; Li, Y.; Vincent, M. P.; Krier-Burris, R. A.; Qian, Y.; Yuk, S. A.; Almunif, S.; Bochner, B. S.; Qiao, B.; Scott, E. A. Controlled adsorption of multiple bioactive proteins enables targeted mast cell nanotherapy. *Nature Nanotechnology* **2024**, *19*, 698-704.

(20) Carrow, K. P.; Hamilton, H. L.; Hopps, M. P.; Li, Y.; Qiao, B.; Payne, N. C.; Thompson, M. P.; Zhang, X.; Magassa, A.; Fattah, M.; Agarwal, S.; Vincent, M. P.; Buyanova, M.; Bertin, P. A.; Mazitschek, R.; Olvera de la Cruz, M.; Johnson, D. A.; Johnson, J. A.; Gianneschi, N. C. Inhibiting the Keap1/Nrf2 Protein-Protein Interaction with Protein-Like Polymers. *Adv. Mater.* **2024**, *36*, 2311467.

(21) Du, F.; Qiao, B.; Nguyen, T. D.; Vincent, M. P.; Bobbala, S.; Yi, S.; Lescott, C.; Dravid, V. P.; Olvera de la Cruz, M.; Scott, E. A. Homopolymer self-assembly of poly (propylene sulfone) hydrogels via dynamic noncovalent sulfone–sulfone bonding. *Nat. Commun.* **2020**, *11*, 4896.

(22) Jiang, T.; Hall, A.; Eres, M.; Hemmatian, Z.; Qiao, B.; Zhou, Y.; Ruan, Z.; Couse, A. D.; Heller, W. T.; Huang, H.; de la Cruz, M. O.; Rolandi, M.; Xu, T. Single-chain heteropolymers transport protons selectively and rapidly. *Nature* **2020**, *577*, 216-220.

(23) Wei, Y.; Chen, A.; Lin, Y.; Wei, T.; Qiao, B. Allosteric Regulation in SARS-CoV-2 Spike Protein. *Phys. Chem. Chem. Phys.* **2024**, *26*, 6582.

(24) Qiao, B.; Olvera de la Cruz, M. Enhanced binding of SARS-CoV-2 spike protein to receptor by distal polybasic cleavage sites. *ACS Nano* **2020**, *14*, 10616-10623.
